# Supplementary material for: Seasonal variations in the nutritive value of fifteen multipurpose fodder tree species: A case study of north-western Himalayan mid-hills
Source: PLoS One. 2022 Oct 25;17(10):e0276689. doi: 10.1371/journal.pone.0276689 (PMC9595570; doi:10.1371/journal.pone.0276689)
Supplement: S1 File — (DOCX) [file pone.0276689.s001.docx]

**Supporting file – 1 (S1 File)**

**Protocol for chemical analysis followed in current investigation**

**Dry matter per cent** (DM)

The air-dried leaves kept in paper bags were dried at 60±5°C in an oven till a constant weight is achieved and weight was recorded, treatment and replication wise. The loss in weight was considered as the moisture percentage and the moisture percent was calculated by using the formula:

$$Moisture percent=\frac{W_{1}-W_{2}}{W_{1}} x 100$$

Where;

W_1_ = Weight of samples before hot air oven drying

W_2_ = Weight of samples after hot air oven drying

Whereas, Dry matter percent was calculated as:

Dry mater per cent = 100 – Moisture per cent

## Crude protein percent (CP)

One gram dried grinded leaf samples was taken in 500ml Micro- kjeldahl’s flask in which 10 g of digestion mixture (10 g copper sulphate + 20 g ferrous sulphate + 200 g potassium sulphate + 3g HgO + 1g selenium powder) and 20 ml of concentrated sulphuric acid was added. Small pieces of calcium carbonate were added, so as to check bumping. The flask was then kept on the electric heater till the colour of the digested material become light bluish-green. The flask was cooled overnight and the material was diluted with distilled water to make the aliquot of 100 ml in a volumetric flask.

Total nitrogen in the leaves was estimated by Kjeldahl method. The 10 ml of saturated sodium hydroxide (40%) solution was taken in micro Kjeldahl’s flask. Thereafter aliquot (5ml) was added and kept on the electric heater with distillation unit connected through tap to the condenser. The lower end of the condenser was dipped in a solution of 20ml of boric acid (4%) containing mixed indicator in a 250ml beaker. Distillation was carried out for 30 minutes during which all the ammonia released was trapped in the boric acid solution, whichwas then titrated against 0.1N H_2_SO4 to a chocolaty colour end. Total nitrogen was calculated by the standard value, i.e., 1ml of 0.1N H_2_SO4 = 0.0014 mg of nitrogen.

$$Crude protein \left( \% \right)= \frac{100 x Y x 0.0014 x 6.25}{X x W} x 100$$

Where;

Y= Aliquot volume made of digested sample (100ml); T = Titre value (ml);

X= Volume taken for distillation (5ml);

W = Weight of sample taken for digestion (1g)

## Crude fibre percent (CF)

The sample after ether extraction was transferred from the thimble in to a spoutless beaker of 1litre capacity. Then 25 ml of 2.04 N sulphuric acid solution was added to it and volume was make up to 200 ml with water. The beaker was sealed with the round bottom flask filled with cold water to act as a condenser in order to maintain the volume of the contents of the beaker. The contents were boiled for 30 minutes on heating mantle, then removed and cooled by adding around 200 ml of water to avoid charring of muslin cloth during washing, which results passage of finer particles during filtration and washing. The contents of the flasks were filtered through the muslin cloth. The residue in the cloth was washed with distilled water 2-3 times to remove the acid.

The residue was transferred to the same beaker carefully. Then 25 ml of 2.50 N sodium hydroxide was added to the beaker and make up volume was made to 200 ml with ordinary water. The beaker was sealed with the round bottom flask filled with cold water to act as a condenser for maintaining the volume of the content of the beaker. The contents were boiled for 30 minutes on heating mantle. Later the contents of beaker were filtered through the muslin cloth after addition of around 200 ml water for cooling. Finally, again the residue was washed 2-3 times with distilled water to remove the alkali.

The residue was then transferred to the pre weighed crucible and kept in hot air oven at 100±5°C for drying. The crucible was cooled in desiccator and weighted to a constant weight. The content was ashed in a muffle furnace at 550-600°C for 1-2 hours, cooled in a desiccator and weighted. The loss in the weight during ashing was the percentage of the crude fibre and was calculated as follows:

$$Per cent of Crude fibre = \frac{W_{1}-W_{2}}{W} x 100$$

Where:

W_1_ = weight of crucible plus oven dried residue after acid and alkali digestion

W_2_ = weight of crucible plus ash

W = weight of oven dried sample

## Ether extract percent (EE)

Estimation of ether- extract was done with the help of Soxhlet’s apparatus. 5g oven dried sample was taken in a thimble of Whatman filter paper no. 1 and placed in an extractor. The extractor was connected with pre weighted oil flask below and condenser above.

Petroleum ether of boiling point 60-80°C was poured into the extraction tube with 60 ml more than required for permitting siphon to the oil flask placed on the heater. Cold water was passed through the condenser during the extraction process. Extraction was carried out for 6 hours till the liquid was as clear as clean water. The flask was then disconnected and dried in the hot air oven at 100±5°C for 4-6 hours till the ether was completely evaporated. It was cooled in a desiccator and weighed to a constant weight. The difference in the weight of oil flask after and before extraction denoted the ether extract of the sample and was calculated as follows:

$$Per cent of Ether Extract = \frac{W_{2}-W_{1}}{W} x 100$$

Where;

W = weight of dry sample taken W_1_ = weight of an empty oil flask

W_2_ = weight of oil flask after extraction

**Total ash** (TA)

Five grams of dried sample was taken in a weighted crucible and ashed in a muffle furnace at 550-600°C till the content was free from black particles. Then the crucible was cooled in desiccator and weighted to a constant weight. The ash content was calculated as follows:

$$Per cent of Ash = \frac{W_{2}-W_{1}}{W} x 100$$

W = weight of dry sample taken W_1_ = weight of empty crucible

W_2_ = weight of crucible with ash

## Nitrogen free extract percent (NFE)

Nitrogen free extract is determined by subtracting the percentage of crude protein, ether extract, crude fibre and total ash on dry matter basis. Nitrogen free extract can be calculated as follows:

NFE = 100 – [CP% + EE% + CF% + Ash%]

**Organic matter** (OM)

Organic matter of the sample was calculated by using following formula: OM = CP% + EE% + CF% + NFE%

## Total carbohydrate (TC)

The total carbohydrate of the sample was calculated by adding percentage of crude fibre and the percentage of nitrogen free extract.

TC= TC% + NFE%

## Acid detergent fibre (ADF) Preparation of acid detergent solution (ADS)

Twenty grams of cetyltrimethyl ammonium bromide (CTAB) was dissolved in one litre of 1 N H_2_SO_4_.

## Procedure

Approximately 1 g of sample was taken in a spoutless beaker of 1 L capacity. To this, 100 ml acid detergent solution and 2 ml of decalin were added. The contents were refluxed for exactly 1 hour. After refluxing, the residue was filtered through pre-weighed sintered glass crucible (Grade-I) using vacuum pump, washed with hot water 2-3 times followed by acetone to remove all salts. The crucible containing residue was dried in hot air oven (100 ± 5°C) and weighed again.

The ADF was calculated as follows:

$$ADF \left( \% \right)= \frac{(Weight of crucible with residue-Weight of empty crucible)}{Weight of sample taken} x 100$$

## Neutral detergent fibre (NDF) Preparation of neutral detergent solution (NDS)

EDTA or Disodium ethylene diamino tetra acetate (18.61 g) and 6.81 g of sodium borate decahydrate were put together in a large beaker with some distilled water and heated on hot plate until dissolved. Similarly, 30 g sodium lauryl sulphate was dissolved in distilled water and 10 ml of 2- ethoxyethanol (ethylene glycol monoethyl ether) was added to it. The solution of sodium lauryl sulphate and 2- ethoxyethanol was added to the previous solution. 4.56 g of disodium hydrogen phosphate (anhydrous) was taken in another beaker and some amount of distilled water was added and the contents were heated until dissolved. Then, it was added to solution containing other ingredients and volume was made up to one litre with distilled water.

## Procedure

Leaf sample (0.5 to 2 g) was taken in a 500 ml spout less beaker. To it was added 100 ml preheated Neutral detergent solution (NDS), 0.5g sodium sulphite (anhydrous) and 2 ml of decalin and the contents of spout less beaker were refluxed for an hour after the initial onset of boiling. Then, the contents of beaker were filtered through pre-weighed 50 ml sintered glass crucible (G-I) using oil-free vacuum pump. The contents were washed repeatedly with hot boiling water and then acetone to remove all salts. The sintered crucible containing residue was dried in hot air oven (100 ± 5°C) overnight, cooled and weighed to a constant value. The crucible was kept for ashing in a muffle furnace at 550 ^0^C for 2-3 h and crucible along with ash was weighed again.

$$NDF \left( \% \right)= \frac{(Weight of crucible cell wall constituents-Weight of empty crucible)}{Weight of sample taken} x 100$$

## Estimation of minerals

**Digestion of samples:** Diacid (HNO_3_+ HClO_4_) in the ratio of 4:1 was used for the digestion of plant samples. For this 0.5g of crushed leaf sample was taken and 10-15ml of diacid mixture was added to it. Then the contents were digested by placing on hot up to the point when no sample remained in the conical flask and colourless solution was formed. Final volume was made up to 100ml with distilled water. This extract was stored for further estimation of different minerals (sample extract).

**Phosphorus** (P)

**Preparation of solution:** Ammonium Vanadate- molybdate solution

**Sol. A:** Dissolve Ammonium molybdate (22.5 g) in 400 ml distilled water.

**Sol. B:** Dissolve Ammonium metavanadate (1.25 g) in 300ml of boiling water. Cool the Sol. A and cool the Sol. B

**Ammonium-vanadate-molybdate solution**: Sol. A and Sol. B were mixed, then 250 ml conc. HNO_3_ acid was added and allowed to cooled. Finally, the volume of 1 litre was made up with the addition of distilled water.

**Sample preparation:** Digested sample (5 ml) was taken in 25ml volumetric flask. 5ml of ammonium-vanadate-molybdate solution was added to it. It will be yellow in colour. Final volume of 25ml was made with the addition of distilled water.

**Standard preparation:** Potassium dihydrogen orthophosphate (0.2195 g) was dissolved in distilled water and final volume was made 1 litre, it will be 50ppm (working standard solution). 0.5ppm of standard was made by adding 0.5ml of 50ppm standard solution + 10ml of ammonium-vanadate molybdate solution + double distilled water to make up the volume in 50ml volumetric flask. Similarly, 1ppm, 1.5ppm, 2ppm, 2.5ppm and 3ppm standards were made by using the following formula

Conc. of working Std. × ml of sample extract taken = Conc. of sample × make up volume

**Blank preparation:** For preparing blank solution distilled water + 10ml ammonium vanadate molybdate solution were added in a 25ml volumetric flask.

The spectrophotometer was set at 470nm of wavelength, samples were fitted and absorbance was noted for each sample. Then a graph was plotted between concentration and absorbance for phosphorus. Using the graph, P content of samples was determined.

**Potassium** (K)

**Standard preparation:** Potassium chloride (1.907 g) was dissolved in double distilled water and final volume was made to 1 litre for 1000ppm concentration. 5ml from this solution in 100ml volumetric flask with makeup volume 100ml gave 50 ppm solution. 2, 5, 10 and 20 ml from 50ppm solution were taken in 50ml volumetric flask to obtain 2, 5, 10 and 20ppm solutions by making up volume 50ml.

**Sample preparation:** 5ml of digested sample was taken and final volume was made to 50ml with double distilled water. Readings were noted by using flame photometer.

**Calcium** (Ca)

**Standard preparation:** Calcium carbonate (2.5 g) was dissolved in double distilled water with addition of small quantity of Hydrochloric acid and final volume was made to 1 litre for 1000ppm concentration. By using this 50ppm solution was made by taking 5ml of 1000ppm in 100ml volumetric flask. Then 2ppm, 5ppm, 10ppm, 20ppm solutions were made by taking 2ml, 5ml, 10ml, 20ml of 50ppm solution in 50ml volumetric flask and making up the volume with double distilled water.

**Sample preparation:** Digested sample (5 ml) was taken and final volume was made to 50ml with double distilled water. Readings were noted by using flame photometer

## Copper, Zinc, Iron and Manganese

The digested samples were used for analysis using atomic absorption spectrophotometer for content of micro nutrients Cu, Zn, Fe and Mn using standards prepare for different concentrations. The readings were expressed in parts per million.

## Total Phenols (PHE)

Phenol estimation was done by using Folin- Ciocalteu method

**Sample extract preparation**: Dried 0.2 g leaf powder was taken; 10 ml 50% methanol was added to it and was subjected to centrifugation at 10000 rpm for 20 minutes at 4°C. The extract obtained was stored in plastic bottles in refrigerator (4^0^C) for further analysis.

**Standard curve**: This was prepared by dissolving 25mg tannic acid in 25 ml distilled water and then diluted to 1:10 in distilled water. Different ml of this solution was taken, to this was added 2.5ml of 20% sodium carbonate and 0.5ml 1N folin-ciocalteu reagent and uniform make up volume done with distilled water. These were read at 725nm in spectrophotometer and absorbance noted down. Standard curve of absorbance to concentration was plotted.

**For sample analysis**: Methanolic leaf extract (0.1 ml) was taken to it was added 2.5ml 20% sodium carbonate and 0.5ml 1N folin-ciocalteu reagent and uniform volume was made up. The samples were then read at 725 nm in spectrophotometer and absorbance noted. The ppm of phenol in sample was estimated from standard curve using sample absorbance and later expressed in suitable unit.

## Non-tannin phenols

Non-tannin phenols were estimated after binding the total tannin phenols with insoluble polyvinyl pyrrolidone (polyvinyl polypyrrolidone, PVPP). 1.0 ml of the tannin- containing extract and 1.0 ml of PVPP (1ml containing 100mg PVPP) were added the in the test tube and vortexed. Later the tube was kept at at 4°C for 15 min, vortexed again, then centrifuged for 10 minutes and the supernatant was collected. This supernatant contained only simple phenolics other than tannins (the tannins would have been precipitated along with the PVPP). Then 0.5 or 1 ml of supernatant was taken in the test tube to it was added 2.5ml 20% sodium carbonate and 0.5ml 1N folin ciocalteu reagent. The samples were then read at 725 nm in spectrophotometer and absorbance noted. The ppm of non-tannin phenols in sample was estimated from standard curve using sample absorbance and later expressed in suitable unit.

## Total tannin phenols (TAN)

Total tannin phenols of the sample were calculated by using following formula:

Total tannin phenols = Total Phenols – Non tannin phenols

**Nitrates** (NO_3_)

**Sample extract:** Leaf powder (1 g) was taken in 20 ml distilled water and heated on water bath for 20 minutes, cooled and filtered through Whatman no. 1 filter paper. The extract was stored in cold storage at 4°C till further analysis. For accuracy analyse the samples at the earliest after extraction.

## Standards Stock solution 0.25 g/L NO3-N (=250mg/l, 250 ug/ml)

In a 1 litre volumetric flask, 600ml Type-I-water (deionised) was taken then 1.805 g potassium nitrate was added and stirred to dissolve**.** After all KNO_3_ was dissolved, water was added to make up to the mark of 1 litre this makes 250ppm of standard solution. Nitrate standards should be stored at 4°C.

**Blank** – Since leaves are pigmented samples a separate blank was required for each sample. This blank consisted of the leaf extract, 0.8 ml of conc. H_2_SO_4_ (minus salicylic acid) and 19 ml of 2 N NaOH.

## Reagents:

**Salicylic acid- H_2_SO_4_ reagent**: Salicylic acid (5 g) was dissolved in 100 ml of conc. H_2_SO_4_. The salicylic acid-H_2_SO_4_ reagent was made fresh every week and stored in a brown bottle in cold storage.

**2 N NaOH**: NaOH pellets (40 g) were dissolved in 100 ml of water and volume was made up to 500ml was made with Deionised water.

## Procedure

Leaf extract (0.25 ml) was taken in 25 ml volumetric flask, 0.8ml of salicylic acid H_2_SO_4_ reagent was added and mixed thoroughly. After 20 minutes 19ml of 2N NaOH was added to raise the pH above 12.5. Sample was allowed to cool to room temperature. Later the absorbance was read at 410nm. For preparation of standard curve same procedure was used where instead of leaf extracts, standard at various concentrations were used and other reagents were the same. A standard curve was prepared by plotting standard at various concentration against their absorbance. Nitrate concentration in leaf sample was determined from the standard curve using their absorbance.

**Hydrocyanic Acid** (HCN)

HCN content in leaves was estimated by alkaline titration method.

## Reagents-

1. NaOH solution - 0.5g in 20ml water
2. Ammonium hydroxide (6N) – 400ml ammonia hydroxide (liquid ammonia) add distilled water to make up volume 1 litre
3. Potassium iodide (5%) – 5g in distilled water make up volume 100ml
4. Silver nitrate (0.02N) – 3.4g in distilled water make up volume 1 litre

**Procedure**- Ten gram leaf powder was placed in 800ml Kjeldahl flask with 200ml of distilled water the mixture was left to stand for 2-4 hrs with complete connection to distillation apparatus along with receiver flask. After the stipulated time the contents were heated on Kjeldahl assembly and 150-160 ml distillate collected in receiver flask with NaOH solution, later the content was diluted to 250ml with water. From the above 250ml volume, 100ml was taken in conical flask to this 8ml of 6N ammonium hydroxide solution and 2ml of potassium iodide solution was added. These contents were then titrated with silver nitrate filled in in micro burette. The end point was faint but permanent turbidity clearly visible against black background. Blank reading was determined by using water instead of distillate, other reagents and end point colour remaining the same. Subtraction of blank reading from sample reading gave the final reading.

**Calculation:** 1 ml of 0.02N AgNO_3_ = 1.08 mg HCN

**Saponins** (SAP)

Saponin extraction was done using two different solvents. The first solvent, acetone, was used to extract crude lipid from the samples, while the second solvent (methanol) was used for the extraction of the saponin proper, 2.0g of the sample was folded into a thimble and put in a Soxhlet extractor with a reflux condenser fitted on top. Extraction was done with acetone in a 250 cm^3^ capacity round bottomed flask for 3 hours, after which the apparatus was dismantled and another 150 cm^3^ capacity round bottomed flask containing 100 cm^3^ of methanol was fitted to the extractor and extraction was carried on for another 3 hours. The weight of the flask was taken before and after the second extraction in order to make the change in weight. At the end of the second extraction, the methanol was recovered by distillation and the flask was oven-dried to remove any remaining solvent in the flask. The flask was then allowed to cool and the weight of the flask taken.

$$Saponins (\%)=\frac{A-B}{SM} x 100$$

Where, A = mass of flask and extract; B = mass of empty flask; SM =sample mass

## Mimosine

## Reagents:

1. HCl (0.1N)

1. Ferric chloride (0.5%) in 0.1 N HCl
2. Standard mimosine solution (1mg/ml) in 0.1N HCl.
3. Activated charcoal

## Preparation of standard curve

0, 0.1, 0.2, 0.4, 0.6, 0.8 and 1 ml standard mimosine solution was taken in separate flasks for standards and to each of these 0.4 ml ferric chloride was added and final volume was made 10ml with 0.1N HCl. The standards were then read at 535nm on spectronic-20 spectrophotometer and graph of concentration versus absorbance was prepared.

## Sample preparation:

1-1.5g dried leaf powder was taken in beaker, to it 30-40ml 0.1N HCl was added. This was stirred and boiled gently for 5 minutes on hot plate. After cooling this solution was filtered through nylon cloth into a 100ml measuring cylinder. The residue was again boiled by 20-25ml of 0.1NHCl and filtered into the 100ml cylinder. The procedure was repeated again. Finally, the volume of the filtrate in measuring cylinder was made up to 100ml using 0.1N HCl**.**

## Sample analysis

Blank was prepared with 10 ml of above filtrate, which was taken in conical flask and to it 10 ml of 0.1N HCl was added along with a pinch of activated charcoal. This was shaken for 5 minutes and then filtered through Whatman No. 1or 2 filter paper. If the filtrate was not clear again activated charcoal was added and it was filtered.

Sample was prepared by taking 1ml of sample filtrate with 8.6ml 0.1N HCl and 0.4ml of Ferric chloride and read at 535nm in spectrophotometer. The ppm of mimosine in sample was estimated from standard curve using sample absorbance and later expressed in suitable unit.
