# Supplementary material for: Seasonal variations in the nutritive value of fifteen multipurpose fodder tree species: A case study of north-western Himalayan mid-hills
Source: PLoS One. 2022 Oct 25;17(10):e0276689. doi: 10.1371/journal.pone.0276689 (PMC9595570; doi:10.1371/journal.pone.0276689)
Supplement: S1 Table — (DOCX) [file pone.0276689.s002.docx]

## S2 Table. Month of palatability trial for fodder tree species.

| **Multipurpose tree species (MPTs)** | **Month of palatability trail** |
| --- | --- |
| *C. australis, P. floribundum, Q. glauca, Q. Leucotrichophora* | May |
| *A.catechu, A.chinensis, B. variegata, G. optiva* | December |
| *F. roxburghii, L. leucocephala, O. glandulifera, O. oojeinensis* | January |
| *M. composita, M. serrata, S. tetrasperma* | September |
