# Supplementary material for: Seasonal variations in the nutritive value of fifteen multipurpose fodder tree species: A case study of north-western Himalayan mid-hills
Source: PLoS One. 2022 Oct 25;17(10):e0276689. doi: 10.1371/journal.pone.0276689 (PMC9595570; doi:10.1371/journal.pone.0276689)
Supplement: S2 Table — (DOCX) [file pone.0276689.s003.docx]

## S3 Table. Scheme of establishment of palatability trial.

| **Days** | **Animal Number** | | | | | |
| --- | --- | --- | --- | --- | --- | --- |
|  | **524** | **533** | **541** | **547** | **536** | **554** |
| 1^st^ Day | A | B | A | B | A | B |
| 2^nd^ Day | B | A | B | A | B | A |
| 3^rd^ Day | A | B | A | B | A | B |
| 4^th^ Day | B | A | B | A | B | A |
| 5^th^ Day | A | B | A | B | A | B |
| 6^th^ Day | B | A | B | A | B | A |

Here, A = fodder from first MPT; B = fodder from second MPT
