# Supplementary material for: Seasonal variations in the nutritive value of fifteen multipurpose fodder tree species: A case study of north-western Himalayan mid-hills
Source: PLoS One. 2022 Oct 25;17(10):e0276689. doi: 10.1371/journal.pone.0276689 (PMC9595570; doi:10.1371/journal.pone.0276689)
Supplement: S4 Table — (DOCX) [file pone.0276689.s005.docx]

## S5 Table. Ranking of fodder tree species during summer season for nutritional value.

| **Species** | **Parameters** | | | | | | | | | | | | | | | | | | | | | | **Total** | **Rank** |
| --- | --- | --- | --- | --- | --- | --- | --- | --- | --- | --- | --- | --- | --- | --- | --- | --- | --- | --- | --- | --- | --- | --- | --- | --- |
|  | **DM** | **CP** | **EE** | **CF** | **Ash** | **ADF** | **NDF** | **NFE** | **OM** | **TC** | **P** | **K** | **Ca** | **Cu** | **Fe** | **Mn** | **Zn** | **PHE** | **TAN** | **HCN** | **NO_3_** | **SAP** |  |  |
| ***A. chinensis*** | 6.1 | 10 | 3.3 | -8.9 | 2.7 | -5.3 | -5.9 | 5.1 | 10 | 7.9 | 10 | 6.7 | 1.0 | 8.0 | 9.8 | 1.4 | 0.8 | -1.7 | -4.4 | 0.0 | -0.4 | -5.0 | 51.1 | 10 |
| ***B. variegata*** | 10 | 2.3 | 5.1 | -6.4 | 4.7 | -5.8 | -6.3 | 9.6 | 9.7 | 10 | 8.4 | 9.8 | 2.3 | 9.1 | 10 | 2.1 | 10 | -3.6 | -4.4 | 0.0 | -3.1 | -5.0 | 68.4 | 3 |
| ***C. australis*** | 8.3 | 3.6 | 4.2 | -5.9 | 10 | -5.2 | -6.7 | 7.7 | 8.7 | 8.4 | 7.2 | 5.7 | 10 | 8.7 | 9.9 | 2.5 | 2.9 | -0.7 | -0.5 | -10 | -0.6 | -4.5 | 63.4 | 4 |
| ***F. roxburghii*** | 4.6 | 4.4 | 4.3 | -4.0 | 7.3 | -7.7 | -7.3 | 9.3 | 9.2 | 8.6 | 3.0 | 8.5 | 3.5 | 10 | 10 | 2.1 | 2.6 | -1.6 | -0.7 | 0.0 | -3.2 | -3.1 | 59.7 | 6 |
| ***L. leucocephala*** | 4.7 | 8.7 | 5.3 | -2.8 | 5.4 | -2.4 | -3.2 | 8.6 | 9.5 | 7.6 | 2.4 | 9.3 | 3.1 | 8.7 | 9.8 | 2.2 | 1.4 | -10 | -5.0 | 0.0 | -2.2 | -4.5 | 56.7 | 7 |
| ***M. composita*** | 5.7 | 6.9 | 2.7 | -3.1 | 6.2 | -2.6 | -3.4 | 9.3 | 9.4 | 8.2 | 7.3 | 9.6 | 4.8 | 8.2 | 9.9 | 2.3 | 2.6 | -1.6 | -4.7 | -8.0 | -7.3 | -1.8 | 60.5 | 5 |
| ***M. serrata*** | 4.8 | 6.7 | 6.0 | -3.1 | 7.9 | -4.1 | -4.8 | 8.5 | 9.1 | 7.7 | 5.6 | 8.3 | 2.9 | 8.2 | 9.8 | 2.2 | 2.6 | -0.7 | -1.0 | 0.0 | -3.9 | -0.9 | 71.9 | 1 |
| ***O. glandulifera*** | 7.1 | 3.8 | 3.5 | -4.8 | 4.0 | -6.3 | -10 | 10 | 9.8 | 9.5 | 6.1 | 6.2 | 2.6 | 7.4 | 9.5 | 1.3 | 0.2 | -2.5 | -7.9 | 0.0 | -3.2 | -2.8 | 43.5 | 13 |
| ***O. oojeinensis*** | 6.1 | 5.3 | 3.2 | -8.9 | 5.0 | -5.3 | -5.7 | 6.4 | 9.6 | 8.9 | 5.7 | 8.8 | 2.9 | 7.8 | 9.8 | 1.4 | 1.3 | -1.3 | -2.7 | 0.0 | -2.8 | -3.7 | 51.8 | 9 |
| ***P. floribundum*** | 6.5 | 4.7 | 10 | -4.2 | 5.9 | -5.5 | -6.0 | 8.7 | 9.5 | 8.3 | 5.5 | 10 | 2.5 | 7.8 | 9.9 | 2.1 | 4.2 | -0.6 | -0.4 | 0.0 | 0.0 | -10 | 68.9 | 2 |
| ***Q. glauca*** | 8.3 | 5.3 | 3.4 | -10 | 5.8 | -7.1 | -9.5 | 5.5 | 9.5 | 8.7 | 4.8 | 7.5 | 2.8 | 8.2 | 9.8 | 8.3 | 9.9 | -2.0 | -6.7 | 0.0 | -10 | -3.4 | 49.1 | 11 |
| ***Q. leucotrichophora*** | 8.1 | 2.7 | 4.7 | -7.6 | 2.7 | -10 | -8.8 | 8.8 | 10 | 10 | 2.8 | 7.8 | 1.7 | 7.8 | 9.5 | 10 | 1.4 | -2.9 | -10 | 0.0 | -0.6 | -2.2 | 45.9 | 12 |
| ***S. tetrasperma*** | 7.4 | 4.2 | 5.4 | -4.5 | 5.2 | -7.8 | -8.1 | 9.4 | 9.6 | 9.0 | 6.9 | 6.5 | 3.6 | 6.5 | 9.6 | 3.8 | 0.9 | -3.9 | -0.9 | 0.0 | -1.8 | -4.5 | 56.6 | 8 |

## DM - dry matter (%); CP – crude protein (%); EE – ether extract; CF - crude fiber (%); Ash – total ash (%); ADF - acid detergent fiber (%); NDF - neutral detergent fiber (%); NFE - nitrogen free extract (%); OM - organic matter (%); TC – total carbohydrate (%); P = phosphorus (%); K = potassium (%); Ca - Calcium (%); Cu - copper (ppm); Fe - iron (ppm); Mn - manganese (ppm); Zn - zinc (ppm); PHE - phenol (%); TAN - total tannin (%); HCN - Hydrocyanic acid (mg 100 g^-1^); NO3 - Nitrate (ppm); SAP - saponin content (ppm)
