# Supplementary material for: Seasonal variations in the nutritive value of fifteen multipurpose fodder tree species: A case study of north-western Himalayan mid-hills
Source: PLoS One. 2022 Oct 25;17(10):e0276689. doi: 10.1371/journal.pone.0276689 (PMC9595570; doi:10.1371/journal.pone.0276689)
Supplement: S7 Table — (DOCX) [file pone.0276689.s008.docx]

## Table S8. Nutrient and mineral requirement for dairy cattle (DM basis).

| **Contents** | **Growing cattle** | **Gestating cattle** | **Early**  **lactation cattle** | **Maximum tolerable concentration** |
| --- | --- | --- | --- | --- |
| Dry Matter (%)* | 61.4 | 61.0 | 60.7 | 72.34 |
| Ether extract (%)× | 11.23 | 14.08 | 12.76 | 15.82 |
| Crude Fibre (%)* | 36.08 | 37.28 | 38.48 | 49.81 |
| Crude Protein (%)* | 10.84 | 12.65 | 15.20 | 21.34 |
| Total Ash (%)× | 19.31 | 20.60 | 22.57 | 43.51 |
| Calcium (%)× | 0.31 | 0.31 | 0.30 | 1.5 |
| Copper (ppm)^ | 12 | 15.2 | 15.7 | 40.00 |
| Iron (ppm) ~ | 150 | 118 | 24 | 500 |
| Phosphorus (%)×~ | 0.20 | 0.19 | 0.19 | 1.00 |
| Potassium (%)× | 0.60 | 0.60 | 0.70 | 3.0 |
| Magnesium (%)× | 0.10 | 0.12 | 0.20 | 0.40 |
| Manganese (ppm)^ | 20 | - | 40 | 1000 |
| Zinc (ppm) ~ | 33 | 31 | 63 | 1000 |
| Sodium (%)× | 0.08 | 0.06 | 0.10 | 0.16 |
| Hydrocyanic Acid (%)^#^ | 2.78 | 2.28 | 1.95 | 4.79 |
| Tannin (%)^ | 0.44 | 0.46 | 0.49 | 4.00 |

## *Thorne et al., (1999), × NRC (1989), # NRC (2001), ^Barry and Blaney (1987) ~ NRC (2005)

***** Thorne, P. J.; Subba, D. B.; Walker, D. H.; Thapa, B.; Wood, C. D.; Sinclair, F. I. The basis of indigenous knowledge of tree fodder quality and its implications for improving the use of tree fodder in developing countries. *Anim. Feed Sci. Technol.* **1999**, *81*, 119-131. https://doi.org/10.1016/S0377-8401(99)00048-6

**×** NRC. *Nutrient requirement of domestic cattle*, National Academy of Science, National Research Council: Washington DC, USA, **1989**.

**#** NRC. *Overview of mineral nutrition in cattle: Dairy and beef*, National Academy of Science, National Research Council: Washington DC, USA, **2001**.

**^** Barry, T. N.; Blaney, B. J. Secondary compounds of forages. In: *Nutrition of Herbivores*, Hacker, J. B., Ternouth, J. H., Eds.; Academic Press: Sydney, **1987**; pp. 91-119.

**~** NRC. *Mineral Tolerance of Domestic Animals*, 2nd ed. (rev.); National Academy of Science, National Research Council: Washington DC, USA, **2005**.
