# Supplementary material for: Seasonal variations in the nutritive value of fifteen multipurpose fodder tree species: A case study of north-western Himalayan mid-hills
Source: PLoS One. 2022 Oct 25;17(10):e0276689. doi: 10.1371/journal.pone.0276689 (PMC9595570; doi:10.1371/journal.pone.0276689)
Supplement: Attachment — Submitted filename: PONE-D-22-16121_reviewer.pdf [file pone.0276689.s009.pdf]

PLOS ONE

Seasonal variations in the nutritive value of fifteen multipurpose fodder tree species: a case study of north-western Himalaya mid-hills

--Manuscript Draft--

**Manuscript Number:**	PONE-D-22-16121
**Article Type:**	Research Article
**Full Title:**	Seasonal variations in the nutritive value of fifteen multipurpose fodder tree species: a case study of north-western Himalaya mid-hills
**Short Title:**	Seasonal nutritive value of multipurpose tree species
**Corresponding Author:**	Prashant Sharma, PhD<br>Dr Yashwant Singh Parmar University of Horticulture and Forestry<br>Solan, Himachal Pradesh INDIA
**Keywords:**	anti-nutritional components; mineral composition; cell wall composition; farmers' preference; livestock; palatability; phenols
**Abstract:**	Multipurpose tree species are recognized as an important fodder source for livestock, but their potential remains untapped due to dearth of knowledge about their nutritive value. Therefore, 15 MPTs, i.e., Acacia catechu, Albizia chinensis, Bauhinia variegata, Celtis australis, Ficus roxburghii, Grewia optiva, Leucaena leucocephala, Melia composita, Morus serrata, Olea glandulifera, Ougienia oojeinensis, Pittosporum floribundum, Quercus glauca, Q. leucotrichophora and Salix tetrasperma were evaluated for nutritional characteristics, relative nutritive value index (RNVI), palatability index and farmers' preference on a seasonal basis in north-western Himalayas mid-hills. Most of the nutritive and mineral content decreased as leaves matured with the exception of ether extract, calcium, copper, organic matter and carbohydrate content, while cell-wall constituents and anti-nutritional contents increased. Overall, M. serrata had the highest RNVI in spring and summer, while G. optiva during autumn and winter. Similarly, L. leucocephala had the highest palatability (97.86%), while M. composita (38.47%) the lowest one. Additionally, G. optiva was the most favored MPT for livestock among farmers, while M. composita was the least ones. The outcome of the study will help policy makers, planners and farm managers in establishing large scale plantations of highly nutritious and palatable species, like G. optiva, L. leucocephala, B. variegata, and M. serrata for year-round supply of green leaves and as a supplement to low-quality feed.
**Order of Authors:**	Manasi Rajendra Navale
	D. R. Bhardwaj
	Rohit Bishist
	C. L. Thakur
	Subhash Sharma
	Prashant Sharma, PhD
	Dhirender Kumar
	Massimiliano Probo
**Additional Information:**	
**Question**	**Response**
**Financial Disclosure**<br><br>Enter a financial disclosure statement that describes the sources of funding for the work included in this submission. Review the submission guidelines for detailed	The authors received no specific funding for this work.

Powered by Editorial Manager® and ProduXion Manager® from Aries Systems Corporation

requirements. View published research articles from *PLOS ONE* for specific examples.<br><br>This statement is required for submission and **will appear in the published article** if the submission is accepted. Please make sure it is accurate.<br><br>**Unfunded studies**<br>Enter: *The author(s) received no specific funding for this work.*<br><br>**Funded studies**<br>Enter a statement with the following details:<br>• Initials of the authors who received each award<br>• Grant numbers awarded to each author<br>• The full name of each funder<br>• URL of each funder website<br>• Did the sponsors or funders play any role in the study design, data collection and analysis, decision to publish, or preparation of the manuscript?<br>• **NO** - Include this sentence at the end of your statement: *The funders had no role in study design, data collection and analysis, decision to publish, or preparation of the manuscript.*<br>• **YES** - Specify the role(s) played.<br><br>* typeset	
**Competing Interests**<br><br>Use the instructions below to enter a competing interest statement for this submission. On behalf of all authors, disclose any competing interests that could be perceived to bias this work—acknowledging all financial support and any other relevant financial or non-financial competing interests.<br><br>This statement is required for submission and **will appear in the published article** if the submission is accepted. Please make sure it is accurate and that any funding sources listed in your Funding Information later in the submission form are also declared in your Financial Disclosure	The authors have declared that no competing interests exist.

statement.<br><br>View published research articles from *PLOS ONE* for specific examples.<br><br>**NO authors have competing interests**<br><br>Enter: *The authors have declared that no competing interests exist.*<br><br>**Authors with competing interests**<br><br>Enter competing interest details beginning with this statement:<br><br>*I have read the journal's policy and the authors of this manuscript have the following competing interests: [insert competing interests here]*<br><br>* typeset	
**Ethics Statement**<br><br>Enter an ethics statement for this submission. This statement is required if the study involved:<br><br>• Human participants<br>• Human specimens or tissue<br>• Vertebrate animals or cephalopods<br>• Vertebrate embryos or tissues<br>• Field research<br><br>Write "N/A" if the submission does not require an ethics statement.<br><br>General guidance is provided below. Consult the submission guidelines for detailed instructions. **Make sure that all information entered here is included in the Methods section of the manuscript.**	The current research submission does not require ethics statement. As, the study mainly focused on the anonymous questionnaire based survey about the preferences of fodder trees among the local farmers. Simultaneously, the cows (livestock) were given the regular tree fodders present in the vicinity areas. No clinical trial were carried out on either human or livestock.

Powered by Editorial Manager® and ProduXion Manager® from Aries Systems Corporation

**Format for specific study types**<br><br>**Human Subject Research (involving human participants and/or tissue)**<br>• Give the name of the institutional review board or ethics committee that approved the study<br>• Include the approval number and/or a statement indicating approval of this research<br>• Indicate the form of consent obtained (written/oral) or the reason that consent was not obtained (e.g. the data were analyzed anonymously)<br><br>**Animal Research (involving vertebrate animals, embryos or tissues)**<br>• Provide the name of the Institutional Animal Care and Use Committee (IACUC) or other relevant ethics board that reviewed the study protocol, and indicate whether they approved this research or granted a formal waiver of ethical approval<br>• Include an approval number if one was obtained<br>• If the study involved *non-human primates*, add *additional details* about animal welfare and steps taken to ameliorate suffering<br>• If anesthesia, euthanasia, or any kind of animal sacrifice is part of the study, include briefly which substances and/or methods were applied<br><br>**Field Research**<br><br>Include the following details if this study involves the collection of plant, animal, or other materials from a natural setting:<br>• Field permit number<br>• Name of the institution or relevant body that granted permission	
**Data Availability**<br><br>Authors are required to make all data underlying the findings described fully available, without restriction, and from the time of publication. PLOS allows rare exceptions to address legal and ethical concerns. See the PLOS Data Policy and FAQ for detailed information.	Yes - all data are fully available without restriction

Powered by Editorial Manager® and ProduXion Manager® from Aries Systems Corporation

A Data Availability Statement describing where the data can be found is required at submission. Your answers to this question constitute the Data Availability Statement and **will be published in the article**, if accepted.<br><br>**Important:** Stating 'data available on request from the author' is not sufficient. If your data are only available upon request, select 'No' for the first question and explain your exceptional situation in the text box.<br><br>Do the authors confirm that all data underlying the findings described in their manuscript are fully available without restriction?	
**Describe where the data may be found in full sentences. If you are copying our sample text, replace any instances of XXX with the appropriate details.**<br><br>• If the data are **held or will be held in a public repository**, include URLs, accession numbers or DOIs. If this information will only be available after acceptance, indicate this by ticking the box below. For example: *All XXX files are available from the XXX database (accession number(s) XXX, XXX.*).<br>• If the data are all contained **within the manuscript and/or Supporting Information files**, enter the following: *All relevant data are within the manuscript and its Supporting Information files.*<br>• If neither of these applies but you are able to provide **details of access elsewhere**, with or without limitations, please do so. For example:<br><br>*Data cannot be shared publicly because of [XXX]. Data are available from the XXX Institutional Data Access / Ethics Committee (contact via XXX) for researchers who meet the criteria for access to confidential data.*<br><br>*The data underlying the results presented in the study are available from (include the name of the third party*	All relevant data are within the manuscript and its Supporting Information files.

and contact information or URL).<br>• This text is appropriate if the data are owned by a third party and authors do not have permission to share the data.<br><br>* typeset	
Additional data availability information:	

Manuscript
Click here to access/download;Manuscript;manuscript.docx

Seasonal variations in the nutritive value of fifteen multipurpose fodder tree species: a case study of north-western Himalaya mid-hills

Manasi Rajendra Navale[1], D.R. Bhardwaj[1*], Rohit Bishist[1], C.L. Thakur[1], Subhash Sharma[2], Prashant Sharma[1*], Dhirender Kumar[1], Massimiliano Probo[3]

[1] Department of Silvicuture and Agroforestry, Dr. YSP University of Horticulture and Forestry, Solan, India

[2] Department of Social Sciences, Dr. YSP University of Horticulture and Forestry, Solan, India

[3] Grazing Systems, Agroscope, Posieux, Switzerland

* Corresponding author

Email: bhardwajdr_uhf@rediffmail.com (DRB); prashantsharma92749@gmail.com (PS)

Short title: Seasonal nutritive value of multipurpose tree species

Acknowledgments.

The authors are grateful to the Head of the Department of Silviculture and Agroforestry, Y.S. Parmar, University of Horticulture and Forestry, Solan (HP), India, for providing the necessary facilities during the study.

Abstract

Multipurpose tree species are recognized as an important fodder source for livestock, but their potential remains untapped due to dearth of knowledge about their nutritive value. Therefore, 15 MPTs, i.e., *Acacia catechu*, *Albizia chinensis*, *Bauhinia variegata*, *Celtis australis*, *Ficus roxburghii*, *Grewia optiva*, *Leucaena leucocephala*, *Melia composita*, *Morus serrata*, *Olea glandulifera*, *Ougienia oojeinensis*, *Pittosporum floribundum*, *Quercus glauca*, *Q. leucotrichophora* and *Salix tetrasperma* were evaluated for nutritional characteristics, relative nutritive value index (RNVI), palatability index and farmers' preference on a seasonal basis in north-western Himalayas mid-hills. Most of the nutritive and mineral content decreased as leaves matured with the exception of ether extract, calcium, copper, organic matter and carbohydrate content, while cell-wall constituents and anti-nutritional contents increased. Overall, *M. serrata* had the highest RNVI in spring and summer, while *G. optiva* during autumn and winter. Similarly, *L. leucocephala* had the highest palatability (97.86 %), while M. composita (38.47 %) the lowest one. Additionally, *G. optiva* was the most favored MPT for livestock among farmers, while *M. composita* was the least ones. The outcome of the study will help policy makers, planners and farm managers in establishing large scale plantations of highly nutritious and palatable species, like *G. optiva*, *L. leucocephala*, *B. variegata*, and *M. serrata* for year-round supply of green leaves and as a supplement to low-quality feed.

Introduction

Livestock are global assets and one of major component of agricultural sector enabling many smallholders in India to escape poverty. India has the world's largest livestock population, with 538.8 million livestock in 2019, increased by 4.6 % in comparison to 2012 [1] and contributing to 5.1 and 17.1 per cent of the total gross value added (GVA) and agriculture & allied sector GVA, respectively [2]. Obviously, this trend of rising livestock population has an effect on the kind of fodder resources required to satisfy the nutritional requirements [3]. Indeed, the nutritional health of livestock is crucial for a sustainable production, which depends on food availability, animal nutrient needs, feed nutritional quality, consumption, digestibility [4] as well as feed metabolism [5]. However, in India, either the natural pastures are degraded or have the carrying capacity of less than one livestock unit per hectare per year

[3]. Simultaneously, it becomes difficult to grow forage crops on farms due to exponential increase of human population and demand for food grains [6].

Therefore, the scarcity of conventional feeds for cattle has forced nutritionists to explore for alternative feed sources [7], when forage sources such are legumes are expensive [8]. This is the case of leaf fodder from multipurpose trees species (MPTs), which are widely regarded as emergency feedstuffs [9], especially in the hilly region [10], due to the high protein, soluble carbohydrate, mineral, and vitamin content of their leaves [11,12]. For this reason, MPTs are left deliberately or planted on farm bunds [13], since they can provide versatile products and play a significant role in rural economies [14,15]. Therefore, MPTs have a fair chance of increasing livestock productivity by reducing the gap between demand and availability of green fodder [16]. In addition, MPTs develop deep tap root system, which can maintain the green phytomass late in the season when the herbaceous layer is dry [16,17]. However, the full potential of MPTs' green fodder has not been completely exploited [9] to develop an alternative and healthy non-traditional feed for ruminants that would supply nutritious fodder throughout the year and increase livestock productivity, due to the paucity of adequate knowledge about their nutritive value and palatability.

Concurrently, several variables, including inherent species genotype [18], seasonal variance [19-21], site capacity, edapho-climatic factors [22] and management aspects [23], contribute in determining the nutritional quality dynamics of fodder trees. Among these, temporal fluctuation in the chemical composition and digestibility of tree species may occur as a result of phenological and climatic variations [21]. Thus, knowledge of tree foliage mineral and nutrient fluctuations during the seasons offers considerable potential to provide high-quality ruminant forage [19], by selecting an appropriate harvesting period for specific tree species in a particular agro-climatic region [24]. Such awareness contributes to their proper usage, as well as to the detection of nutrient deficiencies and recommendations for additional livestock requirements [25].

Moreover, indigenous forest trees and shrubs have recently received considerable attention in research [26]. Indeed, local farmers generally have invaluable traditional knowledge of indigenous and exotic MPTs, but little idea about their nutritional values. Therefore, farmer engagement is crucial, as their knowledge and preferences as future users are essential [27]. Additionally, the integration of scientific and farmer knowledge is critical, because it is often not practical to collect the information on the chemical composition of different MPTs on a

consistent basis, due to the large number of fodder trees species used by farmers. Simultaneously, the complementarities between the two information systems will serve as a catalyst for a more integrated approach to evaluate and select the most suitable tree fodder species for their needs [28]. Nevertheless, there is dearth of detailed information on the species used by farmers, their preference for exotic or indigenous MPTs, and the extent to which they are capable of supplementing and improving the nutritional value assessment in the laboratory. This approach will allow researchers to focus effectively and efficiently on farmers' objectives, while also providing them with results they can understand [28].

However, the nutritional value of some commercial MPTs forage has been studied extensively [13,16,29], but scarce data are available on less commercially important but common MPTs, in particular with regard to changes in forage quality due to seasonal variation and subsequent palatability in the north-western Himalayas. Therefore, to fill this knowledge gap, we selected fifteen different MPTs which were harvested during the four seasons. The objective of the work was to assess and compare the proximate and mineral composition, anti-nutritional and cell wall components, relative nutritive value and farmers' preference of the selected trees species.

Materials and methods

Study area

The study area located in the mid-hill zones of the north-western Himalayas in India (30° 51´ N, 76° 11´ E, elevation 1250 m above mean sea level), having undulating and hilly terrain with elevation and depressions, gentle slopes, and south-eastern aspect. The area comes under the subtropical belt, but slightly skewed towards the temperate climate, with temperatures ranging from 1 °C in winter to 37 °C during the summer, with a mean annual temperature of 19.8 °C. The hottest months are May and June, while the coldest months are December and January. The area receives 1100-1150 mm of rain per year, with most of the rain falling during the monsoon season (July and August) and rarely experiencing snowfall [30,31]. The soil is gravelly sandy loam (Order Inceptisol; Typic Eutrochrept) with 62.9 % sand, 22.3 % silt, and 14.8 % clay with neutral soil reaction, low in organic carbon content (0.13-0.19 %), medium in available N (300-360 kg ha^{-1}) and available K content (300-370 kg ha^{-1}), while contains high available P content (40-50 kg ha^{-1}) [32].

Table 1. Description of different MPTs of mid-hills of north-western Himalayan ecosystem with their leaf phenology.

Species	Family	Common name	Nature	Uses	Leaf phenology			
					**Spring**	**Summer**	**Autumn**	**Winter**
Acacia catechu Willd.	Fabaceae	Khair	Deciduous	Fodder, fuel, dye, timber, tannin, gum resin	oldest	leafless	new	old
Albizia chinensis Osbeck. Merr.	Fabaceae	Chinese albizzia	Deciduous / Evergreen	Fodder, fuel, timber, gum resin, erosion control, reclamation	new	old	older	oldest
Bauhinia variegata Linn. Vern	Fabaceae	Kachnar	Deciduous	Fodder, food, fibre, apiculture fuel, dye, timber, tannin, gum resin, medicinal, ornamental	leafless	new	old	leafless
Celtis australis Linn.	Ulmaceae	Khirak	Deciduous	Fodder, fuel, fibre, timber, Nitrogen fixing,	new	old	oldest	leafless
Ficus roxburghii Wall.	Moraceae	Timbal	Evergreen	Food, fodder, stem's latex for cuts & wounds	new	old	older	oldest
Grewia optiva J. R. Drummond ex Burret.	Tiliaceae	Bhimal	Deciduous	Fodder, fuel, fibre, timber	oldest	leafless	new	old
Leucaena leucocephala (Lam.) De Wit.	Fabaceae	Subabul	Evergreen	Fodder, fuel, fibre, timber, tannin, dye	oldest	new	old	older
Melia composita Wild.	Meliaceae	Darek	Deciduous	Fodder, fuel, timber, medicine, Ornamental, beads and rosaries made from fruit beads	leafless	new	old	leafless
Morus serrata Roxb.	Moraceae	Himalayan mulberry	Deciduous	Food. fibre, fuel, fodder, tannin dye, essential oil, medicine	new	old	oldest	leafless
Olea glandulifera Wall. ex G.Don	Oleaceae	Jharinu	Evergreen	Fodder, species coppices well	new	old	older	oldest
Ougienia oojeinensis (Roxb.) Hochr	Papilionoide ae	Sandan	Deciduous	Fodder, fuel, fibre, timber, host plant for lac insects	leafless	new	old	oldest
Pittosporum floribundum Wight & Arn	Pittosporacea e	Pipalu	Evergreen	Fodder, bark is medicinal contains saponins and pittosporin	older	oldest	new	old
Quercus glauca Thunb	Fagaceae	Bani oak	Evergreen	Fodder, Timber, tannin, medicine, ornamental	oldest	new	old	older
Q. leucotrichophora A.Camus	Fagaceae	Ban	Evergreen	Fodder, Timber, medicine, ornamental	older	oldest	new	old
Salix tetrasperma Roxb.	Salicaceae	Indian willow	Deciduous	Fodder, basket work twigs, construction & planking wood.	new	old	oldest	leafless

Spring: March-April; Summer: May-June; Autumn: September-November; Winter: December-February.

Leaf sampling and pre-processing

Leaves of fifteen MPTs were randomly collected from farm fields (naturally grown on the farm bunds), from March 2014 to February 2015 based on the leaf phenology (Table 1) from all parts of the tree crown (three trees per season per species per replicate). During July and August months (Rainy season) green fodder is available in plenty and easily, therefore tree species are not fed to animals and hence omitted for current evaluation. For each individual MPTs, collected samples were washed, dried (60.5 °C heating air burning until constant weight was obtained), grounded (the Willey mill) and sieved through a 40- mesh sieve in the laboratory for proximate and mineral content analysis.

Chemical analysis

The pre-processed leaves samples were analyzed for the proximate principles [33], i.e., dry matter (DM) (%), crude protein (CP) (%), crude fiber (CF) (%), ether extract (EE) (%), total ash (%), nitrogen free extract (NFE) (%), total carbohydrate (%), organic matter (%), and the cell wall constituent [acid detergent fiber (ADF) and neutral detergent fiber (NDF) (%)] [34-36]. The flame photometer method [37,38] were used to determine the phosphorus (P), potassium (K), and calcium (Ca) content while Atomic-absorption spectrophotometer method [39] for the copper (Cu), iron (Fe), zinc (Zn) and manganese (Mn) content. For mineral analysis, the samples were digested using the diacid (HNO3 + HClO4) in the ratio of 4:1. The total phenol (Folin-Ciocalteau reagent method [40]), tannin [41,42], nitrate [43], hydrocyanic acid (HCN) (talkaline-titration method, [33]) and saponin contents [44] were also assessed. The mimosine content, an alleo-chemical found in the leaves of the *L. leucocephala*, was assessed according to the procedure of Matsumato and Sharman [45].

Relative Nutritional Value Index (RNVI)

A seasonal relative nutritional value index (RNVI) was created to rank the various MPTs in terms of their nutritive value. The MPTs with the highest value of desirable nutritional traits, i.e., CP, EE, total ash, NFE, OM, total carbohydrate, P, K, Ca, Cu, Fe, Mn and Zn, were assigned a score of (+) 10 for a given trait, while the highest values for traits such as CF, ADF, NDF and anti-nutritional traits, i.e., total phenol, tannin, nitrate, HCN and saponin content were given a score of (-) 10. The remaining species were weighted based on the ratio

between their contents and the highest values found for a given trait. The scores obtained by each MPTs were then summed to rank the MPTs.

Palatability analysis

The cafeteria technique suggested by Larbi et al. [46] and later adopted by Mokoboki et al. [47] was used for the palatability analysis at the Dairy farm of the Department of Silviculture and Agroforestry, Dr. YSP university of Horticulture and Forestry, Nauni, India. Six heifers (1-2 years) of Jersey cross breed were chosen and adapted to the selected species by feeding them for about 5 days before starting actual investigation. Two kg of fodder per animal per day from a particular MPT were fed to heifers and on every alternate day, and the same heifer was fed with fodder from another MPT every other day to avoid habituation. This approach was repeated for the six days and for all selected MPTs during their respective fodder production month (S1 Table and S2 table). Furthermore, the animals were fed at 10 a.m., and the final readings for the amount refused were taken after 1 hour. The percent of the fodder taken was determined as follow:

$$\text{Fodder consumed} = (\text{Fodder offered} - \text{Fodder refused}) / (\text{Fodder offered}) \times 100$$

The relative palatability ranks were then assigned to each species based on the percentage of fodder consumed by the heifers.

Farmers' preference

A survey was conducted in the nearby three villages to learn about farmers' preferences for the fodder species. Ten farmers were chosen from each village and asked to rate the fodder species chosen for the current study, as well as their preferred time of fodder harvest.

Statistical analysis

Seasonal variability analysis was performed for the nutritive analysis of selected traits i.e., DM, CP, EE, total ash, NFE, OM, total carbohydrate, P, K, Ca, Cu, Fe, Mn, Zn, CF, ADF, NDF, total phenol, tannin, nitrate, HCN and saponin contents of different MPTs. The data for nutritive parameters and palatability were statistically analyzed using the analysis of variance (ANOVA) of a factorial randomized block design ignoring the missing value, as described by Gomez and Gomez [48]. SAS data analysis package 9.2 was used to test the mean of the treatments for significance at the 5 % level of significance, and graphs were created using

JMP 15.1 and R software v. 4.0.5. In addition to it, a multivariate analysis was carried out to assess the variations in the nutritional and anti-nutritional contents as a function of the 15 MPTs. For this analysis, the palatability, proximate composition, mineral contents, cell wall constituents and anti-nutritional contents for the respective MPTs were analyzed through a principal component analysis (PCA) based on the Pearson correlation coefficient index using the XLSTAT 202.5.1 software.

Results

Proximate composition

The proximate composition of the selected 15 MPTs revealed that there were significant variations among MPTs and during different seasons (Table 2 & 3). The maximum DM content was recorded in *A. catechu* (62.50 %) followed by *Q. leucotrichophora* (59.69 %), and the lowest (34.22 %) in *F. roxburghii*, which was found to be statistically equivalent to *L. leucocephala* (35.02 %). The CP content ranged from the 8.49 % (*A. catechu*) to 19.40 % (*A. chinensis*) with an average of 13.30 %. The ether content averaged 3.87 %, varying from 2.32 (*M. composita*) to 6.55 % (*A. catechu*). The highest mean ash content was recorded in *C. australis* (19.59 %), while lowest was in *A. chinensis* (4.93 %), the average being 10.40 %. The maximum and minimum NFE was observed in *P. floribundum* (60.43 %) and *A. chinensis* (33.49 %), respectively. *A. chinensis* had the highest OM content (95.07 %), whereas the minimum was found in *C. australis* (80.41 %), which also recorded the minimum total carbohydrate content (61.45 %), while the maximum was detected in *Q. leucotrichophora* (81.90 %).

Similar to species effect, seasons also had a significant impact on the nutritional contents of the leaves, with the exception of NFE content (Table 2 & 3). In the autumn season, the maximum dry matter content (53.07 %) was detected, followed by the winter season, while the minimum was in the spring season (41.68 %), however it remained statistically similar to summer season. The highest CP content was recorded in the spring season leaves (15.35 %) and the lowest in winter (10.75 %). While, CF (20.58-28.94 %) and carbohydrate contents (69.77-75.78 %) had an opposite trend. The maximum EE content (3.93 %) was recorded in the winter, while the minimum was in summer season (3.38 %) leaves. The ash content increased from spring (9.19 %) to autumn (11.31%) and decreased during winter season (lowest value, 8.75%). The OM content was maximum and minimum during winter (91.25 %) and autumn (88.82 %), respectively.

Table 2. Proximate analysis of MPT leaves in relation to season of harvesting (n=36).

Multipurpose Tree Species (MPTs)	Dry matter content (%)					Crude protein content (%)					Carbohydrate content (%)					Ether extract (%)				
	S_1	S_2	S_3	S_4	Mean	S_1	S_2	S_3	S_4	Mean	S_1	S_2	S_3	S_4	Mean	S_1	S_2	S_3	S_4	Mean
A. catechu	61.44	-	63.32	62.72	62.50^{h}	6.50	-	11.08	7.87	8.49^{a}	74.66	-	76.58	74.97	75.40^{efg}	7.53	-	5.15	6.96	6.55^{g}
A. chinensis	27.90	38.56	48.22	58.73	43.35^{c}	28.00	26.25	13.42	9.92	19.40^{f}	63.11	66.95	79.06	84.26	73.34^{cde}	4.02	2.37	2.25	1.95	2.65^{ab}
B. variegata	-	63.23	52.72	34.73	50.23^{e}	-	11.08	9.04	6.12	8.75^{a}	-	84.80	78.99	77.23	80.34^{gh}	-	3.64	5.13	5.34	4.70^{ef}
C. australis	33.55	52.26	58.33	-	48.05^{de}	27.42	9.33	9.92	-	15.56^{de}	52.97	71.21	60.17	-	61.45^{a}	3.51	3.02	3.66	-	3.40^{bc}
F. roxburghii	35.65	29.07	35.44	36.73	34.22^{a}	12.83	11.50	11.96	10.50	11.70^{abc}	65.09	73.44	64.87	70.00	68.35^{bc}	2.39	3.06	6.26	3.23	3.74^{cd}
G. optiva	52.87	-	57.62	55.63	55.37^{f}	14.0	-	22.5	21.0	19.16^{ef}	73.11	-	65.23	65.27	67.97^{f}	2.83	-	1.77	3.59	2.73^{ab}
L. leucocephala	32.77	29.74	42.92	34.64	35.02^{a}	21.00	22.75	17.33	16.33	19.35^{ef}	65.09	64.63	68.01	69.54	66.82^{b}	3.58	3.81	5.96	3.99	4.34^{de}
M. composita	-	36.23	48.13	-	42.18^{c}	-	18.08	14.00	-	16.04^{def}	-	69.75	83.29	-	69.01^{bcd}	-	1.93	2.71	-	2.32^{a}
M. serrata	42.58	30.42	44.97	-	39.32^{b}	15.17	17.50	12.25	-	14.97^{cd}	59.79	65.13	71.13	-	65.35^{ab}	4.08	4.34	4.56	-	4.33^{de}
O. glandulifera	52.76	44.85	59.59	47.17	51.09^{e}	14.58	9.92	9.32	8.17	10.50^{ab}	73.53	81.10	78.66	80.62	78.48^{efgh}	3.18	2.49	2.27	3.56	2.87^{abc}
O. ojeinensis	-	38.50	51.64	48.80	46.31^{d}	-	14.00	13.42	10.50	12.64^{bcd}	-	75.53	71.91	75.26	74.23^{de}	-	2.29	3.53	3.69	3.17^{abc}
P. floribundum	34.52	41.16	58.86	36.95	42.87^{c}	11.08	8.75	14.58	12.25	11.67^{abc}	70.69	70.90	76.41	78.08	74.02^{def}	4.78	7.19	3.47	3.90	4.84^{ef}
Q. glauca	51.10	52.58	56.74	59.18	54.90^{f}	7.58	14.00	9.92	8.75	10.06^{ab}	79.90	73.99	77.11	77.91	77.23^{efgh}	6.99	2.47	4.79	6.76	5.25^{f}
Q. leucotrichophora	58.47	51.04	63.41	65.85	59.69^{g}	8.17	7.00	12.25	10.50	9.48^{ab}	84.14	85.14	78.23	80.10	81.90^{h}	1.05	3.40	4.36	5.15	3.49^{bcd}
S. tetrasperma	16.52	46.78	54.17	-	39.16^{b}	14.00	11.08	9.92	-	11.67^{ab}	75.21	76.45	70.24	-	73.96^{a}	3.17	3.90	4.00	-	3.69^{cd}
Mean	41.68^{a}	42.65^{a}	53.07^{c}	49.19^{b}		15.35^{b}	14.17^{b}	12.66^{a}	10.75^{a}		69.77^{a}	73.77^{bc}	72.33^{ab}	75.78^{c}		3.93^{b}	3.38^{a}	3.99^{b}	4.38^{b}	
$CD_{0.05}$	MPTs = 2.69; S = 1.39; MPTs × S = 1.78					MPTs= 3.82; S= 1.97; MPTs × S = 2.55					MPTs = 5.02; S = 2.59; MPTs × S = 3.35					MPTs = 0.89; S = 0.46; MPTs × S = 0.59				

S_1- Spring (March-April); S_2- Summer (May-June); S_3- Autumn (September-November); S_4- Winter (December-February).

Means followed by different letters are significantly different ($P<0.05$)

Table 3. Proximate analysis of MPT leaves in relation to season of harvesting (n=36).

Multipurpose Tree Species (MPTs)	Total ash content (%)					Nitrogen free extract (%)					Organic matter content (%)				
	S_1	S_2	S_3	S_4	Mean	S_1	S_2	S_3	S_4	Mean	S_1	S_2	S_3	S_4	Mean
A. catechu	11.31	-	7.18	10.20	9.56^{bcd}	50.07	-	57.85	52.43	53.45^{ef}	88.69	-	92.82	89.80	90.44^{def}
A. chinensis	4.87	4.43	5.28	5.16	4.93^{a}	29.78	31.48	34.15	38.55	33.49^{a}	95.13	95.57	94.72	94.84	95.07^{g}
B. variegata	-	7.76	6.68	6.34	6.98^{ab}	-	59.39	49.13	45.89	51.47^{cde}	-	92.24	93.16	93.66	93.02^{fg}
C. australis	16.10	16.43	26.25	-	19.59^{g}	35.57	47.59	35.89	-	39.68^{ab}	83.90	83.57	73.75	-	80.41^{a}
F. roxburghii	19.69	12.00	16.91	16.27	16.22^{f}	50.51	57.54	44.38	48.21	50.16^{cde}	84.31	88.00	83.09	83.73	83.78^{b}
G. optiva	9.71	-	10.51	9.83	10.02^{cde}	54.86	-	43.65	47.80	48.77^{cde}	89.92	-	89.49	90.17	89.86^{cd}
L. leucocephala	10.33	8.81	8.52	10.14	9.45^{bc}	53.94	53.41	54.02	51.09	53.11^{def}	89.67	91.19	91.48	89.96	90.55^{def}
M. composita	-	10.23	14.81	-	12.52^{e}	-	57.44	53.72	-	55.58^{ef}	-	89.77	84.99	-	87.38^{c}
M. serrata	20.96	13.03	12.06	-	15.35^{f}	47.57	52.95	54.80	-	51.77^{cde}	79.04	86.97	87.94	-	84.65^{b}
O. glandulifera	8.71	6.49	9.75	7.65	8.15^{bc}	60.48	62.04	57.34	57.98	59.46^{ef}	91.29	93.51	90.25	92.35	91.85^{def}
O. ojeinensis	-	8.18	11.14	10.54	9.95^{bcd}	-	39.92	44.76	48.81	44.50^{bcd}	-	91.82	88.86	89.46	90.05^{def}
P. floribundum	9.95	9.66	9.03	9.27	9.48^{bcd}	56.95	54.27	64.93	65.56	60.43^{ef}	90.05	90.34	90.97	90.73	90.52^{def}
Q. glauca	5.52	9.54	8.18	6.59	7.46^{b}	36.32	34.06	37.46	36.63	36.12^{a}	94.48	90.46	91.82	93.42	92.55^{efg}
Q. leucotrichophora	6.64	4.39	7.36	4.25	5.66^{a}	55.60	54.84	42.84	22.25	43.88^{bc}	93.36	95.54	94.84	95.75	94.87^{g}
S. tetrasperma	7.62	8.57	15.84	-	10.68^{de}	58.71	58.36	47.99	-	55.02^{ef}	92.38	91.43	84.16	-	89.32^{c}
Mean	10.95^{b}	9.19^{a}	11.31^{b}	8.75^{a}		49.19	51.02	48.19	46.83		89.02^{a}	90.80^{b}	88.82^{a}	91.25^{b}	
$CD_{0.05}$	MPTs= 2.51; S= 1.30; MPTs × S = 1.68					MPTs = 7.45; S = NS; MPTs × S = 4.96					MPTs= 2.54; S= 1.31; MPTs × S = 1.69				

S_1- Spring (March-April); S_2- Summer (May-June); S_3- Autumn (September-November); S_4- Winter (December-February).

Means followed by different letters are significantly different ($P<0.05$)

Mineral composition

The mineral composition showed a significant (P<0.05) variation amongst different MPTs (Table 4 & 5). The maximum P content was recorded in *B. variegata* and *M. serrata* leaves, each displaying identical values (0.25 %), whereas the minimum P content was found in *A. catechu* (0.03 %) leaves. Similarly, K and Ca contents also showed wide variation from 0.98 % (*S. tetrasperma*) to 2.18 % (*M. serrata*) and 10.53 % (*C. australis*) and 1.66 % (*A. chinensis*), respectively. The Cu and Fe contents showed less but significant variations ranging from 16.91 ppm (*M. serrata*) to 22.94 ppm (*A. catechu*) and 504.38 ppm (*O. glandulifera*) to 701.27 ppm (*A. chinensis*) (Table 5), respectively. *Q. glauca* had the highest Mn content (264.99 ppm), while *O. oojeinensis* (33.32 ppm) had the lowest. The minimum Zn content was detected in *A. catechu* (4.29 ppm), while the maximum was in *S. tetrasperma* (56.29 ppm) and thus displayed a huge variation amongst MPTs.

Seasonal variations also had a significant (P<0.05) effect on the mineral composition of the studied parameters, excepting K (Table 4 & 5). The maximum P (0.20 %), Ca (4.90 %) and Zn (31.69 ppm) content was recorded in autumn season, and the minimum P (0.09 %) and Zn (13.90 ppm) in winter season leaves. The highest Cu (22.89 ppm) and Fe (699.13 ppm) contents were recorded during winter season, while the lowest Cu (16.54 ppm) and Fe (653.84 ppm) contents were observed in summer and autumn, respectively. The K content did not change significantly with the seasons. However, the Mn content was the highest in spring (89.96 ppm) and the lowest in summer (60.51 ppm).

Cell wall composition and anti-nutritional contents

There were significant differences (P<0.05) among MPTs for the cell wall constituents, i.e., ADF, NDF and CF contents (Table 6). Q. glauca had the highest ADF (43.79 %) and NDF (61.13 %), while L. leucocephala had the lowest ADF (12.18 %) and NDF (21.51 %). Similarly, *Q. glauca* had the maximum CF content (41.11 %), while *M. composita* had the minimum (13.43 %). Likewise, the anti-nutritional contents also varied significantly (P<0.05) among the different MPTs (Table 6 & 7). The total tannin content varied from 0.57 % (*M. serrata*) to 6.09 % (*Q. glauca*). The HCN contents ranged from 0.0 - 0.08 mg 100 g^{-1} DM under different MPTs. *M. composita* and *C. australis* recorded the maximum (15.85 ppm) and minimum (0.84 ppm) nitrate content, respectively. The saponin contents ranged from 5.40 % (*G. optiva*) to 27.16 % (*P. floribundum*), whereas phenol contents varied from 1.50 % (*M. serrata*) to 15.36 % (*L. leucocephala*).

Table 4. Mineral composition of MPT leaves in relation to season of harvesting (n=36).

**Multipurpose Tree Species (MPTs)**	**Phosphorus content (%)**					**Potassium content (%)**					**Calcium content (%)**					**Copper content (ppm)**				
	**S_1**	**S_2**	**S_3**	**S_4**	**Mean**	**S_1**	**S_2**	**S_3**	**S_4**	**Mean**	**S_1**	**S_2**	**S_3**	**S_4**	**Mean**	**S_1**	**S_2**	**S_3**	**S_4**	**Mean**
A. catechu	0.01	-	0.05	0.02	0.03^{a}	1.52	-	0.93	1.23	1.22^{abc}	5.05	-	3.66	4.70	4.47^{de}	25.90	-	18.40	24.53	22.94^{h}
A. chinensis	0.12	0.25	0.12	0.05	0.13^{bcd}	1.25	1.30	1.60	1.03	1.29^{a}	0.76	1.25	2.08	2.55	1.66^{a}	18.40	16.13	20.23	26.23	20.25^{ef}
B. variegata	-	0.21	0.41	0.12	0.25^{f}	-	1.90	1.20	0.00	1.03^{ab}	-	2.75	1.95	2.55	2.42^{ab}	-	18.40	26.23	22.70	22.44^{gh}
C. australis	0.18	0.18	0.29	-	0.22^{ef}	1.60	1.10	1.18	-	1.29^{abc}	9.28	11.93	10.38	-	10.53^{i}	14.00	17.50	21.00	-	17.50^{ab}
F. roxburghii	0.15	0.08	0.17	0.05	0.11^{bc}	1.10	1.65	1.60	1.80	1.54^{abc}	7.78	4.20	4.78	8.08	6.21^{g}	11.43	20.23	21.90	22.70	19.07^{cde}
G. optiva	0.23	-	0.21	0.17	0.20^{def}	1.30	-	1.83	2.23	1.79^{abc}	6.05	-	4.15	2.63	4.28^{de}	15.63	-	21.83	21.03	19.50^{de}
L. leucocephala	0.15	0.06	0.30	0.07	0.15^{cde}	1.78	1.80	1.97	2.25	1.95^{abc}	3.20	3.75	3.73	5.68	4.09^{de}	16.60	17.60	22.83	21.80	19.71^{e}
M. composita	-	0.18	0.26	-	0.22^{ef}	-	1.85	0.68	-	1.26^{abc}	-	5.75	9.75	-	7.75^{h}	-	16.63	18.40	-	17.52^{ab}
M. serrata	0.50	0.14	0.11	-	0.25^{f}	2.33	1.60	2.62	-	2.18^{c}	3.25	3.43	5.35	-	4.01^{de}	14.93	16.60	19.20	-	16.91^{ab}
O. glandulifera	0.06	0.15	0.16	0.17	0.14^{cd}	1.60	1.20	1.83	1.80	1.61^{abc}	3.08	3.15	4.68	3.71	3.65^{cd}	15.67	14.90	23.63	26.20	20.10^{ef}
O. ojeinensis	-	0.14	0.28	0.11	0.18^{cdef}	-	1.70	1.68	1.05	1.48^{abc}	-	3.48	5.48	5.32	4.76^{e}	-	15.70	17.60	21.00	18.10^{abc}
P. floribundum	0.10	0.14	0.24	0.07	0.14^{bcd}	2.05	1.93	2.55	1.93	2.11^{bc}	3.78	2.98	3.10	4.38	3.56^{cd}	14.83	15.73	21.07	21.80	18.36^{bcd}
Q. glauca	0.05	0.12	0.05	0.03	0.06^{ab}	1.31	1.45	0.65	1.40	1.20^{abc}	2.95	3.33	3.21	2.33	2.95^{bc}	13.10	16.60	19.23	22.70	17.91^{abc}
Q. leucotrichophora	0.02	0.07	0.23	0.12	0.11^{b}	1.13	1.50	1.33	1.23	1.30^{abc}	2.73	2.00	2.88	3.78	2.85^{bc}	13.07	15.70	21.00	21.07	17.71^{ab}
S. tetrasperma	0.10	0.17	0.24	-	0.17^{cdef}	0.90	1.25	0.78	-	0.98^{a}	2.43	4.35	8.30	-	5.03^{f}	31.43	13.23	19.20	-	21.29^{fg}
Mean	0.14^{b}	0.15^{b}	0.20^{c}	0.09^{a}		1.49	1.56	1.49	1.45		4.19^{a}	4.03^{a}	4.90^{b}	4.15^{a}		17.08^{a}	16.54^{a}	20.78^{b}	22.89^{c}	
$CD_{0.05}$	MPTs = 0.08; S = 0.04; MPTs × S = 0.05					MPTs= 1.12; S= NS; MPTs × S = 0.75					MPTs= 1.01; S= 0.52; MPTs × S = 0.67					MPTs = 1.22; S = 0.63; MPTs × S = 0.81				

S_1- Spring (March-April); S_2- Summer (May-June); S_3- Autumn (September-November); S_4- Winter (December-February).

Means followed by different letters are significantly different ($P<0.05$)

Table 5. Mineral composition of MPT leaves in relation to season of harvesting (n=36).

Multipurpose Tree Species (MPTs)	Iron content (ppm)					Manganese content (ppm)					Zinc content (ppm)				
	**S_1**	**S_2**	**S_3**	**S_4**	**Mean**	**S_1**	**S_2**	**S_3**	**S_4**	**Mean**	**S_1**	**S_2**	**S_3**	**S_4**	**Mean**
A. catechu	709.27	-	683.60	708.60	700.49^{i}	114.60	-	55.20	104.00	91.27^{i}	1.18	-	9.10	2.60	4.29^{a}
A. chinensis	691.90	671.07	737.70	704.40	701.27^{i}	31.40	27.20	38.67	37.67	33.73^{a}	16.40	5.10	8.80	5.60	8.98^{b}
B. variegata	-	687.80	696.10	687.87	690.59^{g}	-	38.60	24.10	63.60	42.10^{bc}	-	64.60	24.50	12.30	33.80^{g}
C. australis	625.27	679.40	691.93	-	665.53^{c}	38.60	46.90	30.30	-	38.60^{b}	26.67	18.50	12.60	-	19.26^{d}
F. roxburghii	646.10	687.83	691.50	700.20	681.41^{e}	91.60	38.60	73.63	77.60	70.36^{g}	4.33	16.67	5.30	1.17	6.87^{ab}
G. optiva	658.87	-	687.80	696.10	680.92^{e}	77.00	-	36.60	64.60	59.40^{e}	61.40	-	65.70	37.70	54.93^{h}
L. leucocephala	654.53	675.30	712.73	691.90	683.62^{f}	33.47	41.70	44.90	37.70	39.44^{bc}	16.97	9.33	50.30	2.90	19.88^{de}
M. composita	-	683.67	675.47	-	679.57^{d}	-	42.80	25.10	-	33.95^{a}	-	16.90	39.50	-	28.20^{f}
M. serrata	687.80	675.30	729.40	-	697.50^{h}	64.60	41.77	41.37	-	49.24^{d}	12.63	16.67	22.00	-	17.10^{cd}
O. glandulifera	658.60	650.30	0.00	708.60	504.38^{a}	53.20	25.10	38.27	41.77	39.58^{bc}	9.80	1.60	9.60	0.00	5.25^{a}
O. ojeinensis	-	671.07	683.60	691.90	682.19^{e}	-	27.20	38.27	34.50	33.32^{a}	-	8.30	12.30	7.20	9.27^{b}
P. floribundum	650.37	679.43	691.90	696.20	679.48^{d}	41.70	39.70	47.00	44.97	43.34^{c}	18.80	27.20	26.90	54.40	31.83^{g}
Q. glauca	658.57	671.10	704.47	704.40	684.63^{f}	206.80	157.00	578.60	117.57	264.99^{k}	5.10	63.80	10.50	12.10	22.88^{e}
Q. leucotrichophora	654.50	650.30	708.60	700.27	678.42^{d}	258.80	188.10	105.10	109.20	165.30^{j}	13.10	9.10	23.10	16.90	15.55^{c}
S. tetrasperma	600.40	658.67	712.77	-	657.28^{b}	67.70	71.90	56.30	-	65.30^{f}	7.80	5.97	155.10	-	56.29^{h}
Mean	658.01^{b}	672.40^{c}	653.84^{a}	699.13^{d}		89.96^{d}	60.51^{a}	82.23^{c}	66.65^{b}		16.18^{b}	20.29^{c}	31.69^{d}	13.90^{a}	
$CD_{0.05}$	MPTs = 1.33; S = 0.69; MPTs × S = 0.89					MPTs= 4.63; S= 2.39; MPTs × S = 3.29					MPTs= 3.57; S= 1.84; MPTs × S = 2.38				

S_1- Spring (March-April); S_2- Summer (May-June); S_3- Autumn (September-November); S_4- Winter (December-February).

Means followed by different letters are significantly different ($P<0.05$)

Table 6. Cell-wall composition and total tannin content of MPT leaves in relation to season of harvesting (n=36).

Multipurpose Tree Species (MPTs)	Acid detergent fiber content (%)					Neutral detergent fiber (%)					Crude fiber content (%)					Total tannin content (%)				
	S_1	S_2	S_3	S_4	Mean	S_1	S_2	S_3	S_4	Mean	S_1	S_2	S_3	S_4	Mean	S_1	S_2	S_3	S_4	Mean
A. catechu	23.75	-	34.70	28.17	28.87^{e}	51.00	-	39.95	44.11	45.02^{f}	24.59	-	18.73	22.54	21.95^{b}	12.07	-	9.95	11.73	11.25^{g}
A. chinensis	21.20	22.46	23.52	25.94	23.28^{cd}	32.30	34.37	39.88	48.81	38.84^{d}	33.33	35.48	44.91	45.71	39.86^{d}	5.91	2.27	4.30	0.59	3.27^{d}
B. variegata	-	24.63	28.37	33.07	28.69^{e}	-	36.75	41.37	46.50	41.54^{e}	-	25.41	29.86	31.35	28.87^{c}	-	2.25	3.47	4.23	3.32^{d}
C. australis	21.72	22.22	27.87	-	23.94^{cd}	36.63	39.14	41.25	-	39.01^{d}	17.40	23.62	24.29	-	21.77^{b}	3.25	0.25	1.66	-	1.80^{bc}
F. roxburghii	31.73	32.87	35.16	36.23	34.00^{f}	41.25	42.92	50.88	53.63	47.17^{g}	14.58	15.90	20.49	21.79	18.19^{ab}	6.78	0.35	4.13	5.86	4.28^{e}
G. optiva	25.50	-	16.20	23.23	21.64^{c}	43.26	-	25.79	36.12	35.06^{c}	18.23	-	21.57	17.77	19.19^{ab}	4.50	-	0.14	2.61	2.42^{c}
L. leucocephala	14.29	10.30	11.67	12.46	12.18^{a}	23.47	18.59	21.86	22.10	21.51^{a}	11.15	11.22	14.16	18.45	13.75^{a}	1.46	2.55	3.16	0.56	1.93^{bc}
M. composita	-	11.11	14.68	-	12.89^{a}	-	20.00	23.72	-	21.86^{a}	-	12.31	14.55	-	13.43^{a}	-	2.40	4.96	-	3.68^{d}
M. serrata	14.47	17.59	21.27	-	17.77^{b}	24.50	27.93	30.35	-	27.59^{b}	12.22	12.19	16.32	-	13.58^{a}	1.11	0.49	0.10	-	0.57^{a}
O. glandulifera	21.22	26.73	33.27	35.93	29.29^{e}	56.01	58.56	60.55	67.23	60.59^{i}	13.05	19.06	21.32	22.65	19.02^{ab}	2.58	4.04	5.31	6.78	4.68^{e}
O. ojeinensis	-	22.64	27.00	25.79	25.15^{d}	-	33.13	37.09	44.22	38.15^{d}	-	35.61	27.15	26.45	29.74^{c}	-	1.38	2.58	0.78	1.58^{b}
P. floribundum	13.23	23.30	17.37	13.41	16.83^{b}	29.13	34.92	23.00	24.00	27.76^{b}	13.74	16.63	11.49	12.52	13.59^{a}	0.96	0.22	2.93	2.35	1.62^{bc}
Q. glauca	50.33	29.99	46.37	48.47	43.79^{g}	65.08	55.91	61.16	62.35	61.13^{i}	43.59	39.94	39.65	41.28	41.11^{d}	7.06	3.44	9.53	4.33	6.09^{f}
Q. leucotrichophora	38.89	42.43	43.49	44.67	42.37^{g}	50.24	51.66	52.16	54.45	52.13^{h}	28.54	30.30	35.39	57.85	38.02^{d}	6.83	5.12	7.29	4.60	5.96^{f}
S. tetrasperma	21.60	32.94	35.77	-	30.10^{e}	36.08	47.28	51.77	-	45.04^{f}	16.49	18.09	22.25	-	18.94^{ab}	2.42	0.48	1.70	-	1.53^{b}
Mean	25.17^{a}	24.55^{a}	27.50^{b}	29.76^{c}		40.75^{b}	38.55^{a}	40.05^{b}	45.77^{c}		20.58^{a}	22.75^{ab}	24.14^{b}	28.94^{c}		4.60^{c}	1.94^{a}	4.08^{b}	4.04^{b}	
$CD_{0.05}$	MPTs= 2.95; S= 1.52; MPTs × S = 1.96					MPTs = 1.85; S = 0.96; MPTs × S = 1.24					MPTs= 5.81; S= 3.00; MPTs × S = 3.87					MPTs = 0.84; S = 0.43; MPTs × S = 0.56				

S_1- Spring (March-April); S_2- Summer (May-June); S_3- Autumn (September-November); S_4- Winter (December-February).

Means followed by different letters are significantly different ($P<0.05$)

Table 7. Anti-nutritional analysis of MPT leaves in relation to season of harvesting (n=36).

Multipurpose Tree Species (MPTs)	Phenol content (%)					Nitrate content (ppm)					Saponin content (%)					Hydrocyanic acid (mg/100g DM)				
	S_1	S_2	S_3	S_4	Mean	S_1	S_2	S_3	S_4	Mean	S_1	S_2	S_3	S_4	Mean	S_1	S_2	S_3	S_4	Mean
A. catechu	15.33	-	13.08	12.58	13.67^{h}	0.00	-	15.27	0.00	5.09^{c}	27.68	-	22.04	28.06	25.93^{i}	0.00	-	0.06	0.00	0.02^{ab}
A. chinensis	10.13	3.57	5.97	8.97	7.16^{de}	2.70	0.87	6.30	0.00	2.47^{ab}	11.72	15.84	21.38	12.64	15.39^{efg}	0.10	0.00	0.00	0.00	0.03^{b}
B. variegata	-	7.60	11.38	15.18	11.39^{g}	-	6.08	0.00	12.50	6.19^{c}	-	15.15	12.42	14.41	13.99^{cdef}	-	0.00	0.00	0.00	0.00^{a}
C. australis	5.73	1.43	2.75	-	3.30^{ab}	1.33	1.20	0.00	-	0.84^{a}	9.52	13.55	12.21	-	11.76^{cde}	0.10	0.15	0.00	-	0.08^{d}
F. roxburghii	11.21	3.49	6.18	6.84	6.93^{de}	0.00	6.20	9.43	6.13	5.44^{cd}	20.93	9.39	12.41	7.70	$12.61c^{def}$	0.01	0.00	0.00	0.11	0.03^{b}
G. optiva	4.80	-	2.90	4.40	4.03^{bc}	14.53	-	3.13	0.13	5.93^{c}	2.25	-	8.73	5.21	5.40^{a}	0.00	-	0.00	0.00	0.00^{a}
L. leucocephala	10.10	21.23	22.20	7.92	15.36^{h}	3.23	4.28	0.00	0.00	1.88^{ab}	18.10	13.52	15.17	20.71	16.87^{fgh}	0.02	0.00	0.00	0.00	0.01^{a}
M. composita	-	3.41	8.49	-	5.95^{cd}	-	14.20	17.50	-	15.85^{g}	-	5.43	10.88	-	8.16^{abc}	-	0.12	0.00	-	0.06^{a}
M. serrata	2.71	1.42	0.37	-	1.50^{a}	0.00	7.63	7.43	-	5.02^{c}	16.24	2.64	2.17	-	7.02^{ab}	0.02	0.00	0.00	-	0.01^{a}
O. glandulifera	3.52	5.29	6.98	14.31	7.53^{de}	0.00	24.50	2.73	0.00	6.81^{de}	23.36	8.55	17.90	25.00	18.70^{gh}	0.00	0.00	0.00	0.00	0.00^{a}
O. ojeinensis	-	2.70	4.64	3.62	3.65^{abc}	-	5.37	0.00	0.00	1.79^{ab}	-	11.17	24.45	26.41	20.68^{h}	-	0.00	0.00	0.00	0.00^{a}
P. floribundum	3.54	1.32	7.21	5.86	4.40^{bc}	4.99	0.00	0.73	21.43	6.79^{de}	23.22	30.12	22.90	32.40	27.16^{i}	0.00	0.00	0.00	0.00	0.00^{a}
Q. glauca	9.42	4.19	12.68	7.26	8.39^{ef}	0.00	19.33	12.07	0.00	7.85^{e}	4.79	10.10	19.30	24.14	14.58^{cdefg}	0.11	0.00	0.00	0.13	0.06^{c}
Q. leucotrichophora	9.37	6.07	10.26	6.23	7.98^{de}	14.43	1.10	28.03	18.53	15.53^{g}	4.69	6.58	14.12	16.10	10.37^{bcd}	0.00	0.00	0.00	0.00	0.00^{a}
S. tetrasperma	12.76	8.29	10.69	-	10.58^{fg}	27.23	3.50	7.70	-	12.81^{f}	17.07	30.48	13.23	-	20.26^{h}	0.00	0.00	0.00	-	0.00^{a}
Mean	8.22^{a}	5.39^{b}	8.39^{a}	8.47^{a}		5.70^{a}	7.25^{b}	7.36^{b}	5.34^{a}		14.96^{a}	13.27^{a}	15.29^{a}	19.34^{b}		0.03^{c}	0.02^{b}	0.00^{a}	0.02^{b}	
$CD_{0.05}$	MPTs = 2.31; S = 1.19; MPTs × S = 1.54					MPTs= 1.42; S= 0.74; MPTs × S = 0.95					MPTs = 4.43; S = 2.29; MPTs × S = 2.95					MPTs= 0.02; S= 0.01; MPTs × S = 0.02				

S_1- Spring (March-April); S_2- Summer (May-June); S_3- Autumn (September-November); S_4- Winter (December-February).

Means followed by different letters are significantly different ($P<0.05$)

The maximum ADF (29.76 %), NDF (45.77 %), and saponin contents (19.34 %) were observed in winter season leaves, while the minimum ADF content was detected during summer season. Similarly, the minimum phenol contents were recorded in summer (5.39 %). The highest tannin (4.60 %) and HCN contents (0.03 mg/100 gm) were detected in spring, while the lowest tannin and HCN contents were recorded in summer (1.94 %) and in autumn (0.00 mg 100 g^{-1}), respectively. The nitrate contents in MPTs' leaves showed an increasing trend from spring (5.70 ppm) to autumn (7.36 ppm) and then declined significantly in winter season (5.34 ppm). In *L. leucocephala*, the mimosine content followed an irregular pattern, with the highest levels (1.22 %) recorded in the oldest leaves (spring season) and the lowest levels (0.80 %) in the youngest leaves (winter season) (Fig 1).

Fig 1. Mimosine content (%) in Leucaena leucocephala during different seasons. S1- Spring (March-April); S2- Summer (May-June); S3- Autumn (September-November); S4- Winter (December-February). Means followed by different letters are significantly different (P<0.05). The error bar signifies standard error of mean.

Relative Nutritional Value Index (RNVI) and relative palatability

The seasonal nutritional analysis revealed that *M. serrata* was the most nutritious MPT in the spring and summer seasons (Fig 2), while *A. chinensis* was the least nutritious in the spring and *O. glandulifera* in the summer season (S3 Table - S6 Table). Similarly, *G. optiva* was the most nutritious MPT and *Q. glauca* was the least nutritious in both the autumn and winter seasons. Simultaneously, the palatability of MPTs' leaves varied with the fodder tree species (Table 8). *L. leucocephala* was the most palatable one (97.86 %) and followed by the other species in the order: *B variegata* > *G. optiva* > *M. serrata* > *A. catechu* > *P. floribundum* > *O. oojeinensis* > *C. australis* > *O. ferruginea* > *S. tetrasperma* > *F. roxburghii* > *A. chinensis* > *Q. glauca* > *Q. leucotrichophora* > *M. composita* (38.47 %).

Fig 2. Ranking of 15 selected fodder tree species during different season for relative nutritional value index.

Table 8. Relative palatability and ranking of different fodder tree species.

**Species**	**Days of Feeding (D)**							**Rank**
	**D_1**	**D_2**	**D_3**	**D_4**	**D_5**	**D_6**	**Mean**	
A. catechu	94.67	95.17	94.67	95.67	94.67	95.33	95.03^{e}	5
A. chinensis	85.00	80.83	78.33	86.67	81.67	85.00	82.92^{cd}	12
B. variegata	94.17	98.33	98.33	98.33	96.67	95.83	96.94^{e}	2
C. australis	91.67	90.00	90.00	90.00	90.00	91.67	90.56^{de}	8
F. roxburghii	80.83	88.33	79.17	86.67	78.33	88.33	83.61^{cd}	11
G. optiva	96.67	97.50	96.67	95.00	95.00	93.33	95.69^{e}	3
L. leucocephala	97.50	98.00	97.50	98.50	97.67	98.00	97.86^{e}	1
M. composita	45.83	54.17	22.50	41.67	29.17	37.50	38.47^{a}	15
M. serrata	98.33	95.00	95.83	94.17	95.00	93.33	95.28^{e}	4
O. glandulifera	91.83	88.33	78.33	85.00	83.33	78.33	84.19^{cd}	10
O. ojeinensis	91.67	91.67	93.33	93.33	94.17	92.50	92.78^{e}	7
P. floribundum	95.00	94.00	93.33	91.67	92.50	93.67	93.36^{e}	6
Q. glauca	86.67	51.67	86.67	93.33	72.50	77.50	78.06^{bc}	13
Q. leucotrichophora	63.33	65.00	61.67	79.67	80.00	77.50	71.19^{b}	14
S. tetrasperma	75.00	80.00	95.00	93.33	76.67	86.67	84.44^{cd}	9
**Mean**	85.88	84.53	84.09	88.20	83.82	85.63		
**$CD_{0.05}$**	S = 7.57; D = non-significant; S×D = non-significant							

Means followed by different letters are significantly different (P<0.05)

Principal component analysis (PCA)

The spatial representation of the different nutritive and anti-nutritive values through PCA demonstrated the distinctness of different MPTs (Fig 3). The principal axes 1 and 2 obtained in the analysis accounted for the 32.55 and 15.74 % of total variation, respectively (cumulative value = 48.29 %). The PCA classified the various nutritive and anti-nutritive values into three major groups, i.e., group-I Mn, CF, ADF, NDF, DM, Tannin, group-II Carbohydrate, OM, EE, PHE, Cu, SAP; and group-III Ca, Ash, CP and P while, the remaining parameters and palatability were quite distant. Q. glauca and Q. leucotricophora showed the highest contents of the group-I nutritive parameters, and low K and Zn contents. *L. leucocephala* and *P. floribundum* shared the common characteristics and a high K and NFE contents. *A. catechu*, *B. variagta* and *O. glandulifera* were rich in the group–II nutritive parameters but low in group-III nutritive parameters, while the opposite was detected for *M. serrata*, *C. australis* and *F. roxburghii*, which were rich in group-III nutritive parameters.

Figure 3. Principle component analysis showing the palatability, proximate composition, mineral contents, cell wall constituents and anti-nutritional contents in 15 MPTs. DM - dry

matter (%); CP – crude protein (%); EE – ether extract; CF - crude fiber (%); Ash – total ash (%); ADF - acid detergent fiber (%); NDF - neutral detergent fiber (%); NFE - nitrogen free extract (%); OM - organic matter (%); CAR –carbohydrate (%); P = phosphorus (%); K = potassium (%); Ca - Calcium (%); Cu - copper (ppm); Fe - iron (ppm); Mn - manganese (ppm); Zn - zinc (ppm); PHE - phenol (%); TAN - total tannin (%); HCN - Hydrocyanic acid (mg $100g^{-1}$); NO3 - Nitrate (ppm); SAP - saponin content (ppm); Palatab – palatability.

Farmers' preference

Farmer preference rating of fifteen MPTs of mid-hill ecosystem of north-western Himalayas is presented in Table 9. Amongst different MPTs farmer preferred *G. optiva* the most, followed by *B. variegata* > *M. serrata* > *P. floribundum* > *O. oojeinensis* > *C. australis* > *L. leucocephala* > *Q. glauca* > *Q. leucotrichophora* > *A. catechu* > *A. chinensis* > *F.roxburghii* > *S. tetrasperma* > *O. glandulifera* and *M. composita*. Farmers preferred to harvest the leaves of *A. catechu, B. variegata, F. roxburghii, G. optiva, O. oojeinensis* and *P. floribundum* as fodder during the winter season, while *A. chinensis, C. australis, Q. glauca* and *Q. leucotrichophora* are fed to animal during the summer season. *M. serrata* and *S. tetrasperma* are harvested and offered to livestock during the spring season, whereas *L. leucocephala* could be harvested and fed to animals at any time of year. However, most of the farmers did not feed *M. composita* and *O. glandulifera* to their livestock regularly, so optimum harvesting time could not be ascertained.

Table 9. Preferred ranking and harvesting time for the fodder species by farmers for animal feeding.

Species	Ranking	Harvesting time
A. catechu	10	November- February
A. chinensis	11	April-May
B. variegata	2	November – December
C. australis	6	April – May
F. roxburghii	12	September or November-December
G. optiva	1	November- February
L. leucocephala	7	Year around when available
M. composita	15	-
M. serrata	3	March-November
O. glandulifera	14	December-February
O. ojeinensis	5	May-December
P. floribundum	4	November- February

Q. glauca	8	April-May
Q. leucotrichophora	9	April-May
S. tetrasperma	13	May-June

Discussion

Proximate and mineral composition

The present research demonstrated that the nutritional values and palatability of the fifteen different MPTs investigated varied significantly among themselves and on a seasonal basis. The values obtained for most of the proximate composition parameters evaluated were consistent with those obtained in prior studies [16, 49-53]. However, there are still discrepancies between the present and previous literature values, owing to the intrinsic genotype [18], edapho-climatic factors [22], management regimes [23], and other factors. Similarly, seasonal changes influence the composition of forage nutrients, which has an effect on the feed intake, digestibility, and energy released by farm animals after consumption [54].

Specifically, except for the CF, the majority of the proximate and mineral parameter content of the MPTs increased as leaves matured [12], which is consistent with the current investigation. The DM was the highest during the autumn season (53.07 %), which is in accordance with Gonzalez-Garcia and Archimede [55]. The CP contents of MPTs foliage was comparable to previous studies [56-58] and declined as the season proceeded from summer to winter, i.e., from younger to mature leaves. This may be attributed to the dilution effect, which happens when nutrients (particularly N) are redistributed to other plant parts at the end of the growth cycle [24]. Moreover, except for *A. catechu*, *B. variegata*, and *Q. leucotrichophora*, all MPTs possessed a CP content higher than 10 %, which is beneficial for rumen fermentation [59]. The EE content of the leaves was found to be considerably lower (2-6 %) than the optimum requirement (Table S-5) and previous studies (up to 7.60 %) [9]. Furthermore, contrarily to Shaheen et al. [60], in the present research, the ash content varied according to the species and corroborated well with earlier findings [9, 50, 61], but it was not influenced by seasonality. MPTs under investigation contained an average OM concentration around 90 %, similarly to previous findings [16, 62]. The carbohydrate concentrations ranged from 61-82 percent and increased with leaves, which in divergence with the observations of Singh & Todaria [24], which can be owed to variance in NFE and CF contents.

In the present study, the mineral compositions varied significantly with the variation in the species and season. These differences in mineral composition among the tree species can be

owed to differences in agro-climatic zones, maturity level, genetic makeup, season, soil fertility and harvest technique [50]. The P content (0.03-0.25 %) in the present study was consistent with the level reported by Ganai et al. [9]; Singh et al. [50]; Gonzalez-Garcia et al. [55]. The highest P concentration occurred during the autumn season, which is comparable to the values reported earlier [5, 24,25 63]. However, Ca content in the present study was found to be higher than that reported in the literature (1.95-6.31 % Ca) [50,64-66] and increased as the growing season progressed [67] or at leaf maturity [68]. For this reason, the Ca: P ratios in the present study were considerably wide and varied from 1:16 to 1:149, compared to 2:1 indicating efficient utilization [69], thus highlighting P deficiency in most of the MPTs of the mid-hill Himalayan ecosystem.

Contrary to previous findings [25, 65,70], the K contents in the present investigations did not vary significantly across seasons. The Cu contents in *A. catechu, A. chinensis, B. variegata, L. leucocephala, M. composita, M. serrata, O. glandulifera, O. oojeinensis, P. floribundum*, and *Q. glauca* increased with time, which might be related to copper immobility in plants [71]. The decrease in Cu contents of *G. optiva*, *Q. leucotrichophora*, and *S. tetrasperma* with the maturity of leaves can be explained by the dilution effect caused by the rapid growth of their leaves [72]. Similar to Ca content, Fe content in present evaluation (500-700 ppm) was found to be consistent with earlier findings (520-801 ppm by Shinde and Sankhyan [73]; Rawat et al. [74] or higher (133.05-467 ppm) [16,61,75], increased with maturity [67] and peaking in winter season [25,70].

Mn contents in the MPTs in the present study was higher (33.73-264.99 ppm) than previously reported values by other workers (34.10-90.38 ppm [16,61,73]). The zinc contents of *A. catechu, A. chinensis, B. variegata, G. optiva, Q. glauca*, and *Q. leucotrichophora* was observed to decrease with leaves maturity. This may be because Zn can be mobilized from old reserves for photosynthesis, and the decrease in Zn concentrations in later stages may be due to the dilution effect caused by the rapid growth of leaves during this period [72]. Similarly, Yan et al. [76] found that Zn content was higher at the start of the growing season and decreased as the season progressed. Further, MPTs likes, *B. variegata, G. optiva, P. floribundum*, and *S. tetrasperma* were observed to possess sufficient zinc level to fulfil the dietary needs of the dairy cattle (Table S-5), while other species were zinc deficient.

Cell wall composition and anti-nutritional contents

As the season progressed, ADF and NDF levels were found to increase, due to an increase in leaf cellulose, hemicellulose, and lignin contents [4,19]. The ADF content of tree leaves in the present investigation (12-43 %) is consistent with previous worker's values (22-49 %) [29,49,50,57], whereas the NDF was marginally higher (211-61 %) than that stated by Khan et al. [56], Singh et al. [50], Muhammad et al. [77], and Mhaiskar [78] for different fodder species (24.26-41.77 %). Similar to ADF and NDF contents, the CF content increased from young to mature leaves; since CF is composed of the ADF and NDF fractions. Also, Anele et al. [79] observed a general increase in leaf lignification in mature leaves, resulting in an increase in CF content.

Furthermore, chemical compounds, likes phenol, tannin, mimosine, HCN, nitrate and saponin contents were also assessed, as they are known to play an important role in animal health and productivity, either directly or through their metabolic products, or they can diminish nutrient intake, digestion, absorption, utilization, and produce ill consequences [80]. The values of the phenol content in the current study (1 - 13 %) were consistent with the concentrations reported in *A. nilotica* (16.2 %), *B. variegata* (4.8 %), *O. oojeiuealis* (4.2 %) and *L. leucocephala* (4.9 %) [7], *Celtis africana* (1.4 %) [81] and *Quercus* spp. (7-10 %) [62]. Similarly, the tannin content was also comparable with Rana et al. [7] (2.1-14.6 %), Adeduntan and Oyerinde [82] (0.3-053 %), and Raju et al. [83]. Under the season effect, the phenol and total tannin contents increased with leaf maturity in *A. catechu*, *B. variegata*, *G. optiva*, *L. leucocephala*, *M. composita*, *O. glandulifera*, and *O. oojeinensis*, except in *A. chinensis*, *C. australis*, *F. roxburghii*, *M. serrata*, *P. floribundum*, and *Q. glauca*. This variation in species effect may be a result of physiological behavior and genetic makeup, leading to differential seasonal changes in their phenol and tannin contents [84]. The increase in phenol contents under different species during the winter may be a defensive mechanism against herbivorous insect attacks. Similarly, the increased tannin content in spring may be due to tannin condensation during the winter season, as this is an adaptive mechanism of frost resistant mesophyll cells designed to avoid injury during unfavorable temperate conditions [85] and to protect newly emerging leaves from herbivorous insect attacks.

The HCN concentrations measured in our fodder samples (up to 0.08 mg $100g^{-1}$) are lower than those documented by various researchers (0.03-2.14 mg $100g^{-1}$ [5, 82, 86]. Seasonal variation in the nitrate content of the MPTs leaves may be assigned to increased nitrate absorption from the soil during the active growing season when temperatures were higher. However, the saponin contents (5-27 %) were higher than those observed in *L. leucocephala* (5.8 %) by Aye and Adegun [87]. Additionally, *L. leucocephala* leaves contains a non-protein

amino acid called mimosine, which gets converted into dihydroxypyridone in rumen and can cause excessive salivation, hair loss, poor growth and swelling thyroid in livestock [16]. The mimosine content in the leaves of L. leucocephala was found to be vary from 0.80-1.22 %, which is lower than previously recorded (0.8-2.9 %; [88]). Therefore, in the livestock feed, the *L. leucocephala* leaves should be no more than 30 per cent of total feed on dry mater basis [16].

Further, the majority of the nutritional values in the MPTs of the mid-hills Himalayan fodder tree species were observed to be within the dairy cattle's optimum tolerable concentration range (S7 Table). However, Q. glauca's CF content, especially during the winter season, exceeded the maximum tolerable level for dairy cattle.

Relative palatability and farmers' preference

The highest palatability (%) amongst the studied MPTs based on dry matter intake was observed with *L. leucocephala*, a leguminous tree, while the lowest was observed with *M. composita*, which is in consonance with the observations of Gunasekharan et al [89]. The better palatability of *L. leucocephala* can be attributed to the presence of secondary plant metabolites, such as beta-carotene and xanthophylls [90]. Further, *G. optiva* was observed to be the most nutritious MPT species in both autumn as well as in winter seasons and also the most favored species among farmers as well. *P. floribundum* received a higher farmer ranking and also have high palatability although this species has sporadic occurrence in the region. Farmers favored *Q. glauca* and *Q. leucotrichophora* over *A. chinensis* and *A. catechu*, believing that these trees provided animals with complete contentment.

Conclusions

The present study concludes that there are significant variations among the fifteen different MPTs of the mid-hills north-western Himalayan ecosystem in the proximate and mineral compositions, cell wall constituents, anti-nutrient content, and palatability, which are also influenced by the seasonal effect. Except for EE, Ca, Cu, OM, and carbohydrate contents, the majority of the nutritive contents (CP, total ash, NFE) and mineral composition (P, K, Fe, Mn, Zn) decreased as leaves matured, while cell wall constituents (CF, ADF, NDF) and anti-nutritional content (total phenol, tannin, nitrate, HCN and saponin content) increased. Moreover, the majority of MPTs were found to be high in CP but low in EE. However, there were differences in terms of nutritive value, palatability, and farmer preference. For nutritive

rich fodder for the livestock during the spring season, the preference should be accorded to leaves of *M. serrata, G. optiva, O. glandulifera, P floribundum* and *C. australis*, whereas in summer season *M. serrata, P floribundum, B. variegata* and *C. australi*s are the preferred ones. Similarly, in the autumn season the usage should be shifted toward *G. optiva, L. leucocephala, P floribundum, C australis* and *M. serrata*, while, in winter season *G. optiva, L. leucocephala, P floribundum, O. oojeinensis* and *A. catechu* could be the better choice. Strictly, due to the higher CF content, *Q. glauca* should be ignored for livestock feeding in the winter season. *L. leucocephala* is the most palatable, while *M. composita* was the most unappealing. In addition[illegible] *optiva* is the most preferred MPTs among the farmers of the mid-hills of north-western Himalayan ecosystem, with preferred leaf harvesting during November- February. Thus, MPTs forage harvested at the optimal stage of maturity has significant potential as a source of high-quality forage for livestock, even during critical periods. The finding will help animal nutritionists, policymakers and ecologists to take appropriate measures for the year-round production of nutritive fodder, as well as the conservation and propagation of selected MPTs in sufficient quantity in a variety of agroforestry systems. This will allow their use as supplements to low-quality fodder and straw-based diets in ruminants in order to improve animal health and milk productivity. Indeed, there is urgent need for establishing large scale plantations of highly nutritious and palatable species, likes *G. optiva, L. leucocephala, B. variegata, M. serrata, P. floribundum*, etc. on farmlands, common lands and wastelands. The current study focused exclusively on the nutritive value of the prominent MPTs of the mid-hill Himalayan ecosystem, further investigation is also required to determine how different management practices, such harvesting intensity, could be optimized to produce quality fodder. Simultaneously, more emphasis should be given on nutritive analysis based on trees of different age groups as well as to in-vivo research trials able to identify suitable tree species for livestock production sustainability.

Supporting Information

S1 Table. Month of palatability trial for fodder tree species.

S2 Table. Scheme of establishment of palatability trial

S3 Table. Ranking of fodder tree species during spring season for nutritional value

S4 Table. Ranking of fodder tree species during summer season for nutritional value

S5 Table. Ranking of fodder tree species during autumn season for nutritional value

S6 Table. Ranking of fodder tree species during winter season for nutritional value

S7 Table. Nutrient and mineral requirement for dairy cattle (DM basis).

Acknowledgments

The authors are grateful to the Head of the Department of Silviculture and Agroforestry, Y.S. Parmar, University of Horticulture and Forestry, Solan (HP), India, for providing the necessary facilities during the study.

Author Contributions

Conceptualization: Manasi Rajendra Navale, D.R. Bhardwaj, Rohit Bishist and C.L. Thakur

Formal analysis: Manasi Navale, D.R. Bhardwaj, Subhash Sharma, Prashant Sharma

Investigation: Manasi Rajendra Navale, D.R. Bhardwaj, Rohit Bishist, C.L. Thakur, Subhash Sharma

Methodology: Manasi Rajendra Navale, D.R. Bhardwaj, Rohit Bishist, C.L. Thakur

Visualization: Prashant Sharma

Writing – original draft: Prashant Sharma

Writing – review & editing: Dhirender Kumar, Massimiliano Probo

References

1. DAHD&F. Livestock Census, Department of Animal Husbandry, Dairying & Fisheries, Ministry of Agriculture & Farmers' Welfare, Government of India, New Delhi, India, 2021 [cited 2021 April 30]. Available from: https://www.nddb.coop/information/stats/pop.
2. NAS. National Accounts Statistics-2020, Central Statistical Organisation, Government of India, New Delhi, India, 2021[cited 2021 April 30]. Available from: https://www.nddb.coop/information/stats/GDPcontrib

3. PCI. Report of the sub group III on fodder and pasture management (No. id: 6836). Constituted under the Working Group on Forestry and Sustainable Natural Resource Management. Planning Commission of India. Govt. of India. New Delhi, India, 2011 [cited 2021 April 30]. Available from: https://landportal.org/node/63094
4. Hussain F, Durrani MJ. Nutritional evaluation of some forage plants from Harboi rangeland, Kalat, Pakistan. Pak J Bot. 2009; 41: 1137-1154.
5. Onyeonagu CC, Obute PN. Eze, S.M. Seasonal variation in the anti-nutrient and mineral components of some forage legumes and grasses. Afr J Biotechnol. 2013; 12: 142-149. https://doi.org/10.5897/AJB11.1359
6. Sharma SK. Hortipastoral based land use systems for enhancing productivity of degraded lands under rain fed and partially irrigated conditions. Uganda J Agric Sci. 2004; 9: 320-325.
7. Rana KK, Wadhwa M, Bakshi MPS. Seasonal variations in tannin profile of tree leaves. Asian-Australs J Anim Sci. 2006; 19: 1134 – 1138. https://doi.org/10.5713/ajas.2006.1134.
8. Hamer A, Franzel S, Mounkoro B. Using farmer's criteria to assess profitability of fodder shrubs in the desert margins of West Africa. Land Degrad Dev. 2007; 18: 670-679. https://doi.org/10.1002/ldr.805
9. Ganai AM, Bakshi MPS, Ahmed MA, Matto FA. Evaluation of some top fodder foliage in Kashmir valley. Indian J Anim Nutr. 2009; 26: 142-145.
10. Khatta VK, Tewatia BS, Gupta PC. Nutritional evaluation of tree leaves of humid subtropical region of Himachal Pradesh. Indian J Anim Nutr. 1999; 16: 220-223.
11. Bakshi MPS, Wadhwa M. Tree leaves as complete feed for goat bucks. Small Ruminant Res. 2007; 69: 74-78. https://doi.org/10.1016/j.smallrumres.2005.12.009
12. Azim A, Khan AG, Ahmad J, Ayaz M, Mirza IH. Nutritional evaluation of fodder tree legumes with goats. Asian-Australs J Anim Sci. 2011; 15: 34-37. https://doi.org/10.5713/ajas.2002.34.
13. Khosla PK, Toky OP, Bisht RP, Hamidullah S. Leaf dynamics and protein content of six important fodder trees of the western Himalaya. Agrofor Syst. 1992; 19: 109-118. https://doi.org/10.1007/BF00138501.
14. Sharma P, Singh MK, Tiwari P, Verma K. Agroforestry systems: Opportunities and challenges in India. J Pharmacogn Phytochem. 2017; SP1: 953-957.
15. Sharma P, Singh MK, Tiwari P. Agroforestry: a land degradation control and mitigation approach. Bull Env Pharmacol Life Sci. 2017; 6: 312-317.

16. Datt C, Datt M, Singh NP. Assessment of fodder quality of leaves of multipurpose trees in subtropical humid climate of India. J For Res. 2008; 19: 209-214. https://doi.org/10.1007/s11676-008-0035-2
17. Soni ML, Subbulakshmi V, Yadava ND, Tewari JC, Dagar JC. Silvopastoral agroforestry systems: lifeline for dry regions. In: Dagar JC, Tewari JC, editors. Agroforestry research developments. New York: Nova Publishers; 2016, pp. 245-305.
18. Rubanza CDK, Shem MN, Otsyina R, Bakengesa SS, Ichinohe T, Fujihara T. Polyphenolics and tannins effect on in vitro digestibility of selected Acacia species leaves. Anim Feed Sci Technol. 2005; 119: 129-142. https://doi.org/10.1016/j.anifeedsci.2004.12.004
19. Kokten K, Kaplan M, Hatipoglu R, Saruhan V, Çinar S. Nutritive value of mediterranean shrubs. J Anim Plant Sci. 2012; 22: 188-194.
20. Navale MR, Bhardwaj DR, Bishist R. Seasonal varitation in nutritional quality of *Pittosporum floribundam* in mid hills of Himachal Pradesh. Indian J Anim Nutr. 2017; 34: 233- 237. http://dx.doi.org/10.5958/2231-6744.2017.00040.8
21. Ravetto Enri S, Probo M, Renna M, Caro E, Lussiana C, Battaglini LM, et al. Temporal variations in leaf traits, chemical composition and in vitro true digestibility of four temperate fodder tree species. Anim Prod Sci. 2020; 60: 643-658.
22. Arzani H, Sadeghimanesh MR, Azarniv H, Asadian GH, Shahriyari E. Study of phenological stages effect values of twelve species in Hamadan rangelands. Iran. J Range Desert Res. 2008; 15: 86-95
23. Henkin Z, Ungar ED, Dvash L, Perevolotsky A, Yehuda Y, Sternberg M, et al. Effects of cattle grazing on herbage quality in a herbaceous Mediterranean rangeland. Grass Forage Sci. 2011; 66: 516-525. https://doi.org/10.1111/j.1365-2494.2011.00808.x
24. Singh B, Todaria NP. Nutrients composition changes in leaves of Quercus semecarpifolia at different seasons and altitudes. Ann For Res. 2012; 55: 189- 196.
25. Siulapwa N, Mwambungu A, Mubbunu L, Siyumbi S, Lungu E, Sichilima W. Seasonal variation of nutrients in Hyperrhenia grass from Liempe farm in Lusaka, Zambia. Res J Chem Environ Sci. 2016; 4: 59-67.
26. Roothaert RL, Franzel S. Farmers' preferences and use of local fodder trees and shrubs in Kenya. Agrofor Syst. 2001; 52: 239-252. https://doi.org/10.1023/A:1011896921398
27. Haugerud A. Collinson MP. Plants, genes and people: improving the relevance of plant breeding in Africa. Exp Agric. 1990; 26: 341-362. https://doi.org/10.1017/S0014479700018500

28. Mekoya A, Oosting SJ, Fernandez-Rivera S, Van der Zijpp AJ. Multipurpose fodder trees in the Ethiopian highlands: Farmers' preference and relationship of indigenous knowledge of feed value with laboratory indicators. Agric Syst. 2008; 96: 184-194. https://doi.org/10.1016/j.agsy.2007.08.001
29. Paswan VK, Sahoo A. Feeding of oak (Quercus leucotrichophora) leaves and evaluation for its potential inclusion in the feeding of native heifers of Kumaon Himalaya. Trop Anim Health Prod. 2012; 44: 1931-1938. https://doi.org/10.1007/s11250-012-0159-4
30. Panda S, Bhardwaj DR, Sharma P, Handa AK, Kumar D. Impact of climatic patterns on phenophases and growth of multi-purpose trees of north-western mid-Himalayan ecosystem. Trees For People. 2021; 6: 100143. https://doi.org/10.1016/j.tfp.2021.100143
31. Panda S, Bhardwaj DR, Thakur CL, Sharma P, Kumar D. Growth response of seven multipurpose tree species to climatic factors: A case study from northwestern Himalayas, India. J For Sci. 2022; 68: 83-95. https://doi.org/10.17221/159/2021-JFS
32. Bhardwaj DR, Tahiry H, Sharma P, Pala NA, Kumar D, Kumar A. Influence of Aspect and Elevational Gradient on Vegetation Pattern, Tree Characteristics and Ecosystem Carbon Density in Northwestern Himalayas. Land 2021; 10: 1109. https://doi.org/10.3390/land10111109
33. AOAC. Official Methods of Analysis. 16th ed. Virginia: Association of Official Analytical Chemists; 1995.
34. Goering HK, Van Soest PJ. Forage Fibre Analysis Agricultural Handbook, Washington DC: USDA; 1970.
35. Van Soest PJ. Use of detergents in analysis of fibrous feeds. II A rapid method for determination of fibre and lignin. J Assoc Off Anal Chem. 1963; 46: 829. https://doi.org/10.1093/jaoac/46.5.829
36. Van Soest PJ. Development of a comprehensive system of feed analysis and its application to forages. J Anim Sci. 1967; 26: 119. https://doi.org/10.2527/jas1967.261119x
37. Chapman HD, Pratt PF. Methods of analysis for soils, plants and waters. USA: University of California, Division of Agricultural Sciences; 1961.
38. Jackson ML. Soil Chemical Analysis. New Delhi: Prentice Hall of India; 1987.
39. Kalra YP. Handbook of Reference Methods for Plant Analysis. New York: Soil and Plant Analysis Council, CRC Press; 1998.
40. Singleton VL, Rossi JA. Colorimetry of total phenolics with phosphomolybdic-phosphotungstic acid reagents. Am J Enol Vitic. 1965; 16: 144-158.

41. Makkar HPS, Blummel M, Borowy NK, Becker K. Gravimetric determination of tannins and their correlation with chemical and precipitation methods. J Sci Food Agric. 1993; 61: 161-165. https://doi.org/10.1002/jsfa.2740610205
42. Makkar HPS. Quantification of Tannins in Tree Foliage: a Laboratory Manual. Vienna: FAO/IAEA Working Document; 2000.
43. Cataldo DA, Haroom M, Schrader LE, Young VL. Rapid colorimetric determination of nitrate in plant tissues by nitration of salicylic acid. Commun Soil Sci Plant Anal. 1975; 6: 71-80. https://doi.org/10.1080/00103627509366547
44. Nwinuka NM, Ibeh GO, Ekeke GI. Proximate composition and levels of some toxicants in four commonly consumed spices. J Appl Sci Environ Manage. 2005; 9: 150-155.
45. Matsumoto H, Sherman GD. A rapid calorimetric method for determination of mimosine. Arch Biochem Biophys. 1951; 33: 195-200. https://doi.org/10.1016/0003-9861(51)90098-7
46. Larbi A, Osakwe II, Lambourne JW. Variation in relative palatability to sheep among Gliricidia sepium provenances. Agrofor Syst. 1993; 22: 221-224. https://doi.org/10.1007/BF00705235
47. Mokoboki HK, Ndlovu LR, Malatje MM. Intake and relative palatability indices of acacia species fed to sheep and goats. Agrofor Syst. 2011; 81: 31–35. https://doi.org/10.1007/s10457-010-9352-5
48. Gomez KA, Gomez AA. Statistical Procedures for Agricultural Research. 2nd ed. New York: John Willey and Sons, Inc; 1984.
49. Sultan JI, Rahim I, Nawaz H, Yaqoob M, Javed I. Nutritional evaluation of fodder tree leaves of northern grasslands of Pakistan. Pak J Bot. 2008; 40: 2503- 2512.
50. Singh A, Sharma RK, Barman K, Kumar R. Nutritional evaluation of some promising top foliages. J Res SKUAST-J. 2009; 8: 116-122.
51. Kumar M. Evaluation of Quercus leucotrichophora leaves feeding on growth performance of cross bred heifers. M.Sc. Thesis, Dr. Y. S. Parmar University of Horticulture and Forestry. 2015. Available from: http://krishikosh.egranth.ac.in/handle/1/88429
52. Hashmi MM, Waqar K. Nutritional evaluation of Grewia optiva and Grewia populifolia in different seasons and sites of Chakwal district in Pakistan. Eur Acad Res. 2014; 2: 5047-5057.

53. Bhardwaj DR, Sharma P, Bishist R, Navale MR, Kaushal R. Nutritive value of introduced bamboo species in the north-western Himalaya, India. J For Res. 2019; 30: 2051-2060. https://doi.org/10.1007/s11676-018-0750-2
54. Adesogan AT, Sollenbenger LE, Moore JE. Feeding value and Anti nutritive Factors of Forage tree legume. J Agron. 2006; 7: 174-179.
55. Gonzalez-Garcia E, Caceres O, Archimede H. Nutritive value of edible forage from two Leucaena leucocephala cultivars with different growth habit and morphology. Agrofor Syst. 2009; 77: 131–141. https://doi.org/10.1007/s10457-008-9188-4
56. Khan N, Barman K, Rastogi A, Sharma RK, Yatoo MA. Chemical composition, tannin fractionation and protein binding affinity of some top foliages. Indian J Anim Nutr. 2011; 28: 421-426.
57. Shenkute B, Hassen A, Assafa T, Amen N, Ebro A. Identification and nutritive value of potential fodder trees and shrubs in the mid rift valley of Ethiopia. J Anim Plant Sci. 2012; 22: 1126-1132.
58. Kanta S. Morus serrata, a Himalayan mulberry variety an additional feed for late age silkworm and additional crop in Dhār block of Pathankot district of Punjab. Int J Food Agric Vet Sci. 2013; 3: 82-86.
59. Alam MP, Djajanigara A. Nutritive value and yield of potential tree leaves and shrubs in Bangladesh, In: Procedings of 7th AAAP Animal Science Congress on Sustainable Animal Production and Environmen, Bali, 11-16 July 1994. pp. 317-318.
60. Shaheen G, Khan AR, Khan MJ, Shah, WA, Rehan M, Shahwani NA. Seasonal variations of moisture and ash content in common trees and shrubs used as fodder in Quetta district. Int J Pharm Sci Rev Res. 2016; 38; 36-40.
61. Prakash B, Dhali A, Das KC, Rathore SS, Hazarika H, Rajkhowa C. Nutrient composition and in situ degradability of forest foliages consumed by mithun (Bos frontalis). Anim Nutr Feed Technol. 2008; 8:175-183.
62. Elahi M.Y. Nutritive value of Oak leaves in sheep. Pak J Nutr. 2010; 9: 141-145.
63. Ukanwoko AI, Ironkwe M. Seasonal variation in the nutritive value of some browses fed to goats in Umudike south eastern Nigeria. J Anim Sci Adv. 2013; 3: 13-18.
64. Sheikh GG, Ganie M, Ganie AA. Nutritional evaluation of some tree leaves, feeds and fodders of Ladakh. Indian J Anim Nutr. 2011; 28: 427-431.
65. Badshah L, Hussain F, Perveen S, Sher Z. Seasonal variation in the macromineral in some woody and herbaceous forage in rangeland District, Tank, Pakistan. J Med Plants Res. 2012; 6: 4167-4175.

66. Bahloul N, Kechaou N, Mihoubi N.B. Comparative investigation of minerals, chlorophylls contents, fatty acid composition and thermal profiles of olive leaves (Olea europaea L.) as by-product. Grasas y Aceites. 201; 65: 1-9. https://doi.org/10.3989/gya.0102141.
67. Bowersox TW, Ward WW. Seasonal variation in foliar nutrient concentration of Black Cherry. For Sci. 1977; 23: 429-432. https://doi.org/10.1093/forestscience/23.4.429
68. Negi SS, Pal RN, Ehrick C. Trees fodders in Himachal Pradesh (India). Eshborn: German Agency for Technical Cooperation (GTZ), Federal Republic of Germany; 1980.
69. NRC. Nutrient requirements of sheep. 6th ed. Washington DC: National Academy of Sciences, National Research Council: Washington; 1985.
70. Ayanda K, Beyene ST, Mlambo V, Mopipi K. Mineral concentration and standing crop yield dynamics of forages in semi-arid communal grazing lands of South Africa: effect of landscape and season. Anim Nutr Feed Technol. 2016; 16: 209-226.
71. Fircks YO, Ericsson, T, Sennerby-Forsse L. Seasonal variation of macronutrients in leaves, stems and roots of Salix dasyclados Wimm. grown at two nutrient levels. Biomass Bioenergy 2001; 21: 321–334. https://doi.org/10.1016/S0961-9534(01)00045-9
72. Lambers H, Chapin III FS, Pons TL. Mineral nutrition. In: Lambers H, Chapin FS, Pons TL, editors. Plant Physiological Ecology. New York: Springer; 2008. pp. 255-320.
73. Shinde AK, Sankhyan SK. Mineral contents of locally available feeds and fodders in flood prone eastern plains of Rajasthan and dietary status in ruminants. Anim Nutr Feed Technol. 2008; 8: 35-44.
74. Rawat S, Singh CP, Rawat GS. Chemical analysis of a fodder tree leaves (Milletia auriculata). Asian J Chem. 2009; 21: 4179-4182.
75. Ozkan CO, Atalay AI, Kurt O, Kamalak A. Effect of species on macro and micro mineral composition of oak leaves with respect to sheep requirements. Livest Res Rural Dev. 2016; 28.
76. Yan T, Lü X, Yang K, Zhu J. Leaf nutrient dynamics and nutrient resorption: a comparison between larch plantations and adjacent secondary forests in Northeast China. J Plant Ecol. 2015; 9: 165-173. https://doi.org/10.1093/jpe/rtv034
77. Muhammad IR, Kassah MS, Tanko RJ, Balarabe A. Forage yield of irrigated centre as influenced by stages of maturity in the low – land areas of Zaria, Nigeria. In: Proceeding of the Nigerian Society for Animal Production. Akura, 17-21 March, 2002. pp. 214-215.
78. Mhaiskar RM. Vegetative propagation of Pittosporum floribundum Wight and Arn. through cuttings under mid hill conditions of Himachal Pradesh. M.Sc. Thesis, Dr. Y. S.

Parmar University of Horticulture and Forestry. 2012. Available from: http://krishikosh.egranth.ac.in/handle/1/93425

79. Anele UY, Arigbede OM, Olanite JA, Adekunle IO, Jolaosho AO, Onifade OS, et al. Early growth and seasonal chemical composition of three indigenous multipurpose tree species (MPTS) in Abeokuta, Nigeria. Agrofor Syst. 2008; 73: 89-98. https://doi.org/10.1007/s10457-007-9101-6
80. Akande KE, Fabiyi EF. Effect of Processing Methods on Some Anti-nutritional Factors in Legume Seeds for Poultry Feeding. Int J Poult Sci. 2010; 9: 996-1001, 2010, ISSN 1682-8356.
81. Adedapo AA, Jimoh FO, Afolayan AJ, Masika PJ. Antioxidant properties of the methanol extracts of the leaves and stems of Celtis africana. Rec Nat Prod. 2009; 3: 23-31
82. Adeduntan SA, Oyerinde AS. Evaluation of nutritional and anti-nutritional characteristics of Obeche (Triplochition scleroxylon) and several Mulberry (Morus alba) leaves. Afr J Biochem Res. 2010; 4: 175-178.
83. Raju J, Sahoo B, Chandrakar A, Sankar M, Garg AK, Sharma AK, et al. Effect of feeding oak leaves (Quercus semecarpifolia vs Quercus leucotricophora) on nutrient utilization, growth performance and gastrointestinal nematodes of goats in temperate sub-Himalayas. Small Rumin Res. 2015; 125: 1-9. https://doi.org/10.1016/j.smallrumres.2014.12.013
84. Vaithiyanathan S, Singh M. Seasonal changes in tannin contents of some top feeds in arid region. Indian J Anim Sci. 1989; 59: 1565-1567.
85. Salaj J, Karmulak A. Structural changes in mesophyll cells of Abies alba Mill. during the autumn-spring period. Biologia Bratislava 1995; 50: 93-98.
86. Njidda AA, Olatunji EA, Raji AY. Semi arid browse forages: Their antinutritive substances and in sacco neutral detergent fibre and organic matter degradability. IOSR J Agric Vet Sci. 2012; 1: 21-30.
87. Aye PA, Adegun MK. Chemical Composition and some functional properties of Moringa, Leucaena and Gliricidia leaf meals. Agric Biol J North Am. 2013; 4: 71-77
88. Akingbade AA. Productivity of South African indigenous Nguni goats possessing Synergistesjonesii bacteria on Leucaena leucocephala-grass and natural pastures. Ph.D. Thesis, School of Agricultural Sciences and Agribusiness, University of Natal. 2002. Available from: https://ukzn-dspace.ukzn.ac.za/handle/10413/11737

89. Gunasekharan S, Viswanathan K, Bandeswaran C. Selectivity and palatability of tree fodders in sheep and goat fed by cafeteria method. Int J Sci Environ. 2014; 3: 1767-1771.
90. Meulen US, Struck EA, Harith EC. A review of nutritive value and toxic aspects of Leucaena leucocephala. Trop Anim Prod. 1979; 4: 113-126.

Click here to access/download;Figure;Fig1.tiff

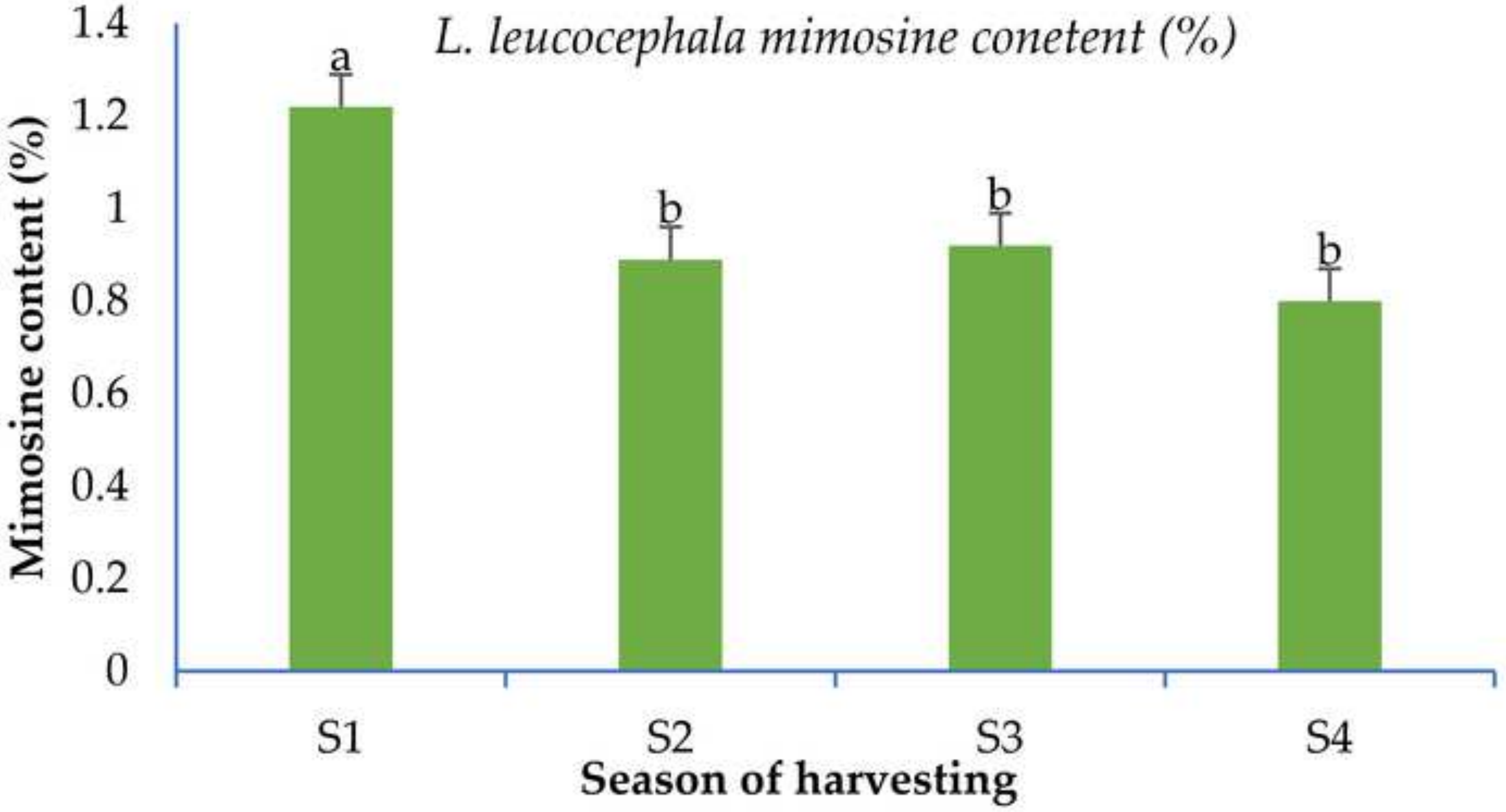

Fig 2

Click here to access/download;Figure;Fig2.tiff

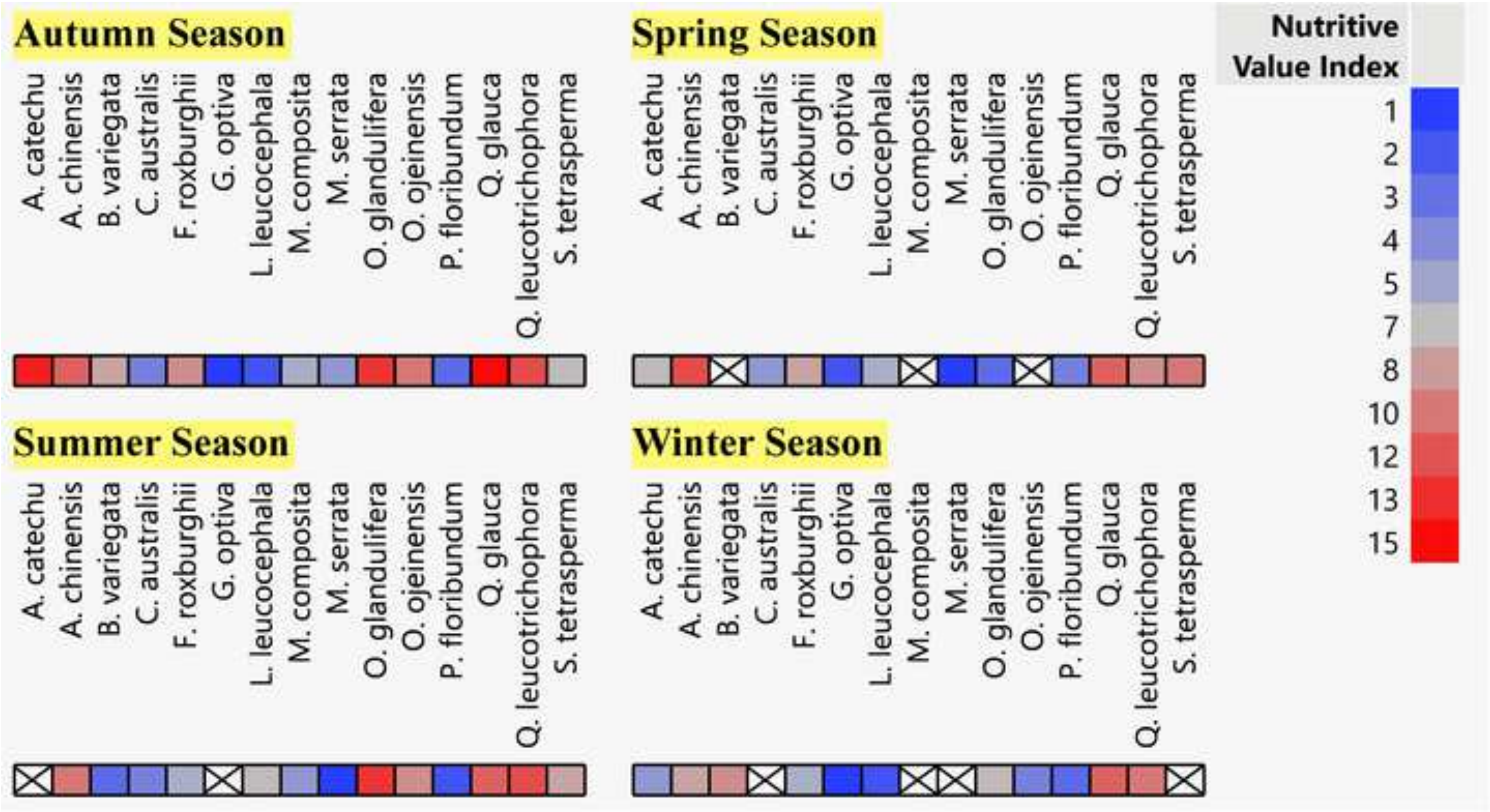

Click here to access/download;Figure;Fig3.tiff

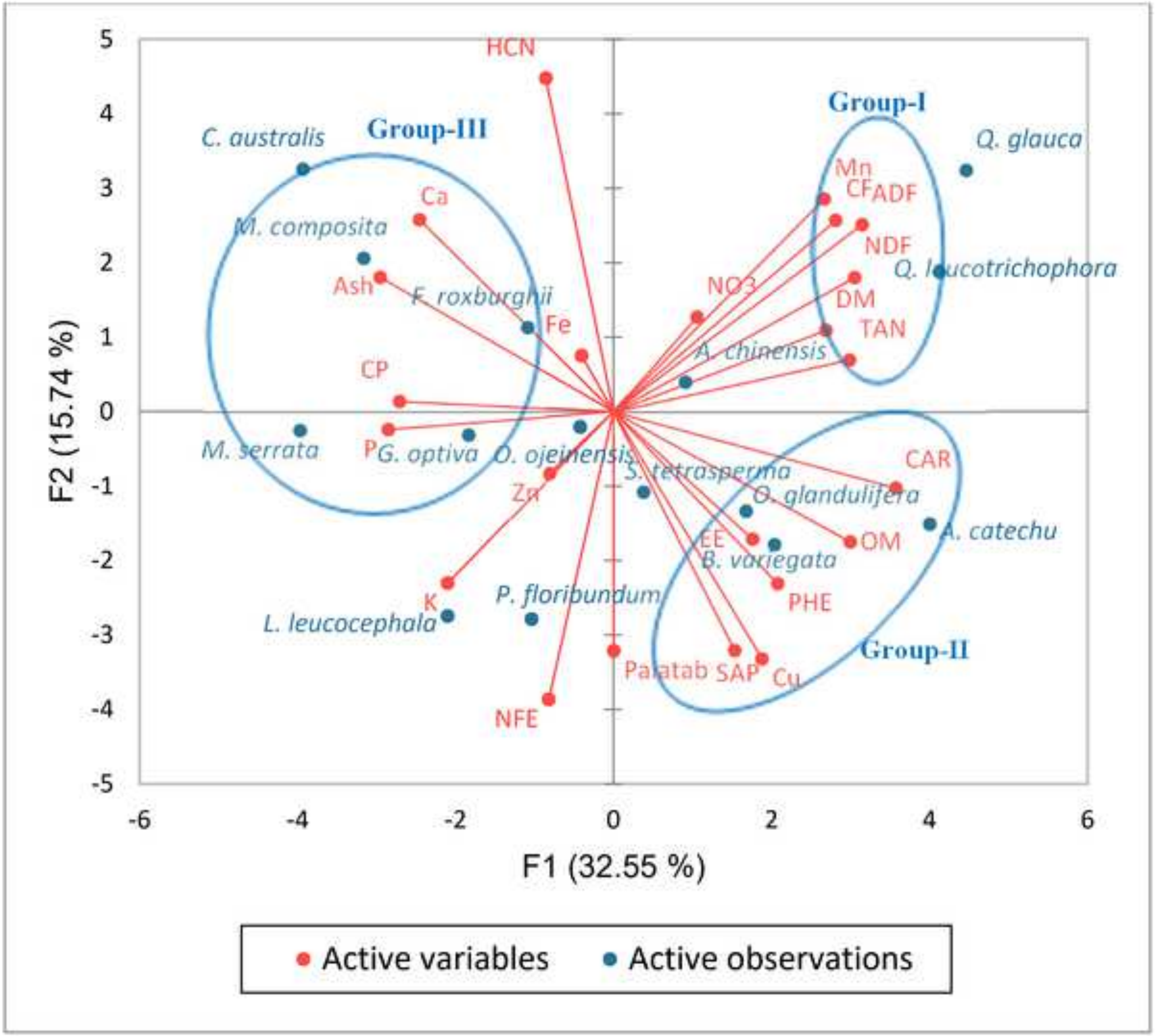

Click here to access/download
Supporting Information
Supporting file.docx
