# Supplementary material for: Seasonal variations in the nutritive value of fifteen multipurpose fodder tree species: A case study of north-western Himalayan mid-hills
Source: PLoS One. 2022 Oct 25;17(10):e0276689. doi: 10.1371/journal.pone.0276689 (PMC9595570; doi:10.1371/journal.pone.0276689)
Supplement: Attachment — Submitted filename: Response to Reviewer.docx [file pone.0276689.s010.docx]

**First of all, we would like to thank the editor and reviewers for their constructive comments to improve the quality of the manuscript. The manuscript has been revised according to the suggestions received. Please find the revised manuscript as track mode change along with point-to-point response as follows: -**

***Editor***

- ***Editor Comment***: Introduction section is too long and can be reduced as also indicated by reviewer.

***Authors Reply***: The Introduction section has been reduced by deleting the following statements:

*However, the full potential of MPTs’ green fodder has not been completely exploited [9] to develop an alternative and healthy non-traditional feed for ruminants that would supply nutritious fodder throughout the year and increase livestock productivity, due to the paucity of adequate knowledge about their nutritive value and palatability.*

*Nevertheless, there is dearth of detailed information on the species used by farmers, their preference for exotic or indigenous MPTs, and the extent to which they are capable of supplementing and improving the nutritional value assessment in the laboratory. This approach will allow researchers to focus effectively and efficiently on farmers' objectives, while also providing them with results they can understand [28].*

- ***Editor Comment***: Much detailed methodology required for chemical investigation section.

***Authors Reply***: We have added a much more detailed description of the methodology used for chemical investigation in ***S1 File. Protocol for chemical analysis followed in current investigation.***

- ***Editor Comment***: Refine the conclusion section.

***Authors Reply***: Conclusion section has been revised thoroughly according to reviewers’ suggestions.

- ***Editor Comment***: Justification of seasonal variation and classification of fodder species according to plant taxonomy will improve discussion.

***Authors Reply***: Compiled as per the comment and following statement added:

*Generally, lower temperature in the winter season has a detrimental effect on the growth of plants. Moreover, the scarce rainfall and other climatic conditions tend to affect the photosynthetic process, resulting in lower forage yield and proximate and mineral composition changes [55]. In addition, in the present, investigation, it has been observed that the leaf phenology also played a major role.*

Regarding the classification of the fodder species according to plant taxonomy, we did not find any significant link to highlight. For instance, *A. catechu* had the lowest crude protein, whereas *A. chinensis* the highest, but both species belong to the family Fabaceae. However, to better clarify, we have added / modified the following statement:

*Globally, many leguminous tree species are used as cattle feed, mostly because of their higher protein content throughout the year [59]. However, in the present study, two leguminous tree species, i.e., A. catechu and B. variegata, along with Q. leucotrichophora, possessed a CP content lower than 10 %, whereas all other fodder tree species had a CP content greater than 10 %, which is beneficial for rumen fermentation [60]. Therefore, despite belonging to the Fabaceae family, A. catechu and B. variegata reported a considerably low CP content, indicating that the proximate composition can largely depend on individual species rather than on family characteristics.*

- ***Editor Comment***: Productivity and yield of fodder species in different seasons should be included.

***Authors Reply***: The average leaf dry biomass yield (kg DM tree^-1^yr^-1^) details have been added in the Table 1.

**Reviewer 1**

- ***Reviewer Comment***: However, authors have to advance discussion part to make findings important for international level and not only for african countries. The analytical values of feedstuffs are adequate to characterize before use.

***Authors Reply***: We try to include in the discussion comments and links to other international important papers important for Asian, European and American countries, even if there is still a limited number of studies on this topic, such as:

Mahieu S, Novak S, Barre P, Delagarde R, Niderkorn V, Gastal F, Emile JC. Diversity in the chemical composition and digestibility of leaves from fifty woody species in temperate areas. Agrofor Syst. 2021; 95: 1295-308. https://doi.org/10.1007/s10457-021-00662-2

Ammar H, López S, González JS, Ranilla MJ. Seasonal variations in the chemical composition and in vitro digestibility of some Spanish leguminous shrub species. Anim Feed Sci Technol. 2004; 115: 327-340. <https://doi.org/10.1016/j.anifeedsci.2004.03.003>

Ravetto Enri S, Probo M, Renna M, Caro E, Lussiana C, Battaglini LM, et al. Temporal variations in leaf traits, chemical composition and in vitro true digestibility of four temperate fodder tree species. Anim Prod Sci. 2020; 60: 643-658. <https://doi.org/10.1071/AN18771>

Kokten K, Kaplan M, Hatipoglu R, Saruhan V, Çinar S. Nutritive value of mediterranean shrubs. J Anim Plant Sci. 2012; 22: 188-194.

Tolera A, Khazaal K, Ørskov ER. Nutritive evaluation of some browse species. Anim Feed Sci Technol. 1997; 67: 181-195. <https://doi.org/10.1016/S0377-8401(96)01119-4>

Gonzalez-Garcia E, Caceres O, Archimede H. Nutritive value of edible forage from two *Leucaena leucocephala* cultivars with different growth habit and morphology. Agrofor Syst. 2009; 77: 131–141. <https://doi.org/10.1007/s10457-008-9188-4>

Fircks YO, Ericsson, T, Sennerby-Forsse L. Seasonal variation of macronutrients in leaves, stems and roots of Salix dasyclados Wimm. grown at two nutrient levels. Biomass Bioenergy 2001; 21: 321–334. <https://doi.org/10.1016/S0961-9534(01)00045-9>

**Reviewer 2**

- ***Reviewer Comment***: Line 122 Rainy -R in lower case

***Authors Reply***: Corrected

- ***Reviewer Comment***: Line 132 was not were

***Authors Reply***: Corrected

- ***Reviewer Comment***: Line 152 Use uniform pattern for YSP

***Authors Reply***: Corrected

- ***Reviewer Comment***: Line 271 *L. leucocephala* italics

***Authors Reply***: Corrected

- ***Reviewer Comment***: Line 300 Delete “The”

***Authors Reply***: Corrected

- ***Reviewer Comment***: Line 465-467, This paragraph is not concluded from the study and hence may be deleted

***Authors Reply***: According to your suggestion, the statement was removed from the manuscript.

- ***Reviewer Comment***: Line 472-476 Conclusion is too long and hence some part may be taken to discussion section

***Authors Reply***: The above statement was removed from the conclusion section and added in the discussion part.
